# The schizophrenia-associated missense variant rs13107325 regulates dendritic spine density

**DOI:** 10.1038/s41398-022-02137-z

**Published:** 2022-09-02

**Authors:** Shiwu Li, Changguo Ma, Yifan Li, Rui Chen, Yixing Liu, Li Pear Wan, Qiuxia Xiong, Chuang Wang, Yongxia Huo, Xinglun Dang, Yongfeng Yang, Luxian Lv, Xi Chen, Nengyin Sheng, Wenqiang Li, Xiong-Jian Luo

**Affiliations:** 1 Key Laboratory of Animal Models and Human Disease Mechanisms of the Chinese Academy of Sciences, Kunming Institute of Zoology, Chinese Academy of Sciences, Kunming, Yunnan 650223 China; 2 Kunming College of Life Science, University of Chinese Academy of Sciences, Kunming, Yunnan 650204 China; 3 State Key Laboratory of Genetic and Resources, Kunming Institute of Zoology, Chinese Academy of Sciences, Kunming, Yunnan 650223 China; 4 Department of Clinical Laboratory, the First Affiliated Hospital of Kunming Medical University, Kunming, Yunnan 650032 China; 5 Department of Pharmacology, and Provincial Key Laboratory of Pathophysiology in Ningbo University School of Medicine, Ningbo, Zhejiang 315211 China; 6 Henan Mental Hospital, the Second Affiliated Hospital of Xinxiang Medical University, Xinxiang, Henan 453002 China; 7 First Department of Neurosurgery, The Second Affiliated Hospital of Kunming Medical University, Kunming, Yunnan 650101 China; 8 Zhongda Hospital, School of Life Sciences and Technology, Advanced Institute for Life and Health, Southeast University, Nanjing, Jiangsu 210096 China; 9 Department of Neurology, Affiliated Zhongda Hospital, Southeast University, Nanjing, Jiangsu 210096 China

**Keywords:** Molecular neuroscience, Physiology

## Abstract

The missense variant rs13107325 (C/T, p.Ala391Thr) in *SLC39A8* consistently showed robust association with schizophrenia in recent genome-wide association studies (GWASs), suggesting the potential pathogenicity of this non-synonymous risk variant. Nevertheless, how this missense variant confers schizophrenia risk remains unknown. Here we constructed a knock-in mouse model (by introducing a threonine at the 393th amino acid of mouse SLC39A8 (SLC39A8-p.393T), which corresponds to rs13107325 (p.Ala391Thr) of human SLC39A8) to explore the potential roles and biological effects of this missense variant in schizophrenia pathogenesis. We assessed multiple phenotypes and traits (associated with rs13107325) of the knock-in mice, including body and brain weight, concentrations of metal ions (including cadmium, zinc, manganese, and iron) transported by SLC39A8, blood lipids, proliferation and migration of neural stem cells (NSCs), cortical development, behaviors and cognition, transcriptome, dendritic spine density, and synaptic transmission. Many of the tested phenotypes did not show differences in SLC39A8-p.393T knock-in and wild-type mice. However, we found that zinc concentration in brain and blood of SLC39A8-p.393T knock-in mice was dysregulated compared with wild-types, validating the functionality of rs13107325. Further analysis indicated that cortical dendritic spine density of the SLC39A8-p.393T knock-in mice was significantly decreased compared with wild-types, indicating the important role of SLC39A8-p.393T in dendritic spine morphogenesis. These results indicated that SLC39A8-p.393T knock-in resulted in decreased dendritic spine density, thus mimicking the dendritic spine pathology observed in schizophrenia. Our study indicates that rs13107325 might confer schizophrenia risk by regulating zinc concentration and dendritic spine density, a featured characteristic that was frequently reported to be decreased in schizophrenia.

## Introduction

Recent large-scale genome-wide association studies (GWASs) have identified hundreds of risk variants for schizophrenia [1–6]. However, elucidating the roles of the identified risk variants in schizophrenia pathogenesis remains a major challenge. Risk variants in coding region play crucial roles in disease pathology [7–13], thus providing critical opportunities to translate the genetic findings into disease mechanism and clinical diagnosis and treatment [14]. Among the schizophrenia GWAS hits, a non-synonymous single nucleotide polymorphism (SNP) rs13107325 (C/T), which encodes SLC39A8 p.Ala391Thr, showed robust association with schizophrenia in schizophrenia GWASs. In 2012, Carrera et al. reported significant association between rs13107325 and schizophrenia [15]. In 2014, Schizophrenia Working Group of the Psychiatric Genomics Consortium (PGC2) validated the strong association between rs13107325 (*P* = 1.54 × 10^−12^) and schizophrenia [5]. In 2018, Pardinas et al. further provided strong genetic evidence for the association between rs13107325 and schizophrenia (*P* = 1.19 × 10^−16^) [16]. Of note, all of these genetic studies reported the consistent risk allele (i.e., T allele) of rs13107325, indicating that rs13107325 is a true risk variant for schizophrenia.

In addition to strong association between rs13107325 and diverse human traits and diseases [17, 18], another intriguing finding is the potential pathogenicity of this missense variant. rs13107325 is located in the eighth exon of *SLC39A8*, which encodes solute carrier family 39 member 8 (ZIP8), a transporter that is responsible for import (uptake) of metal ions cadmium, zinc, manganese, and iron [19, 20]. First, we previously showed that rs13107325 is located in an evolutionary highly conserved coding region, strongly suggesting the functional constraint (functional conservation) of this missense variant [21]. Second, amino sequence analysis showed that the ancestral allele (i.e., C) encodes alanine (Ala) at p391, while the derived (risk) allele T encodes threonine (Thr). Ala is a hydrophobic amino acid, whereas Thr is a hydrophilic amino acid. The physicochemical property difference of Ala and Thr suggests the functionality of this variant. Third, functional annotation using the Combined Annotation Dependent Depletion (CADD) [22–24] indicated that rs13107325 had a CADD score of 34, strongly implying the deleteriousness of rs13107325. Fourth, functional studies supported that rs13107325 is a functional SNP [25]. Zhang et al. showed that rs13107325 affects cellular cadmium accumulation and toxicity [25]. Nakata et al. demonstrated the functionality of rs13107325 in Crohn’s disease by using a knock-in mouse model. They found that SLC39A8-p393T knock-in mice exhibited manganese (Mn) deficiency and increased sensitivity to epithelial injury and pathological inflammation in the colon [26]. Sunuwar et al. also reported that SLC39A8-p393T knock-in mice exhibited abnormal tissue Mn homeostasis and Mn-dependent glycosyltransferase activity in the same year [27]. These lines of evidence support that rs13107325 is a potential pathogenic variant. However, the role and potential pathophysiological mechanisms of this missense variant in schizophrenia remain unknown.

Considering the strong association between rs13107325 and schizophrenia, and the reported functional consequences of this missense variant in cadmium accumulation and Crohn’s disease [25, 26], we hypothesized that rs13107325 may also be a probable pathogenic missense variant for schizophrenia. First, we detailed the association signals (associations with schizophrenia) of the genomic region surrounding rs13107325. Among the risk variants (±250 kb from rs13107325) surrounding rs13107325, rs13107325 showed the most significant association with schizophrenia (Supplementary Figure 1). Of note, only four SNPs are in linkage disequilibrium (LD) with rs13107325 (*r*^2^ > 0.6), and the association *P* values of these SNPs were less than rs13107325. Besides, the Schizophrenia Working Group of the Psychiatric Genomics Consortium identified a credible causal set of SNPs for each of the schizophrenia risk loci, and they reported that association signals of 10 loci were credibly attributable to a known exonic missense variant [5]. Among the 10 missense variants, rs13107325 showed the most significant association with schizophrenia, strongly suggest that rs13107325 is a pathogenic variant for schizophrenia. These convergent lines of evidence suggest the pathogenicity of this missense variant in schizophrenia. Nevertheless, currently we know little about the role and potential pathophysiological mechanisms of this missense variant in schizophrenia. Clearly, functional characterization and biological studies of this missense SNP will not only provide important insights into the pathogenesis of schizophrenia, but also help to identify potential therapeutic target for schizophrenia. Interestingly, the risk allele of rs13107325 only appears in European (8%) and American (5%) populations, but not exists in Asian and African (Supplementary Figure 2) populations. To uncover the role and potential biological implications of this missense variant in schizophrenia, we generated a knock-in mouse model (by introducing a Thr at p393 of mouse SLC39A8 (corresponds to the rs13107325 at p391 of human SLC39A8)) in this study. As the SLC39A8 protein is highly conserved in mouse and human (Supplementary Figure 3), we hypothesized this point-mutation mouse model can provide pivotal information for elucidating the potential pathogenesis of schizophrenia. We firstly investigated the impact of SLC39A8-p393T on global development of the knock-in mice, including body weight, body length, brain weight, and concentrations of metal ions (including cadmium, zinc, manganese, and iron) transported by SLC39A8, in brain and blood of the knock-in mice. We then studied the effect of SLC39A8-p393T on proliferation and migration of neural stem cells (NSCs), as well as cortical development. We next evaluated if the knock-in mouse exhibited behavioral and cognitive abnormalities. We also performed transcriptome analysis to identify the differentially expressed genes (DEGs) in brains of wild-type (SLC39A8-p393A) and knock-in (SLC39A8-p393T) mice. Finally, we investigated the impact of SLC39A8-p393T on dendritic spine density and synaptic transmission, a potential pathology of schizophrenia [28–30]. Our study supports that rs13107325 is a potential pathogenic variant for schizophrenia and indicates that rs13107325 might confer schizophrenia risk by affecting density of dendritic spines.

## Materials and Methods

### Generation of SLC39A8-p393T knock-in mice

Detailed information about generation of SLC39A8-p393T knock-in mice (C57BL/6J background) is provided in Supplementary methods.

### Concentration measurements of cadmium, zinc, manganese, and iron

The cerebral cortices of wild-type (SLC39A8-p.393A) and knock-in (SLC39A8-p.393T) mice were isolated (on ice) from 8-week-old mice brains. Detailed information about concentration measurements of cadmium, zinc, manganese, and iron are provided in Supplementary methods.

### Measurements of blood lipids and ions

The blood samples were obtained from eyeballs of 8-week-old wild-type (SLC39A8-p.393A) and knock-in (SLC39A8-p.393T) mice, then placed into an anticoagulant tube and centrifugated at 1000 rpm for 10 min. The supernatant serum was used to measure the concentrations of glucose, cholesterol, triglycerides, high/low-density lipoprotein, and zinc and calcium ions using cobas® 8000 modular analyzer series (Roche) (with default parameters) at the First Affiliated Hospital of Kunming Medical University with double-blinded. A total of 20 mice were tested (wild-type: *n* = 9; knock-in: *n* = 11).

### Proliferation and migration assays

5-ethynyl-2’-deoxyuridine (EdU) was intraperitoneally injected into the pregnant heterozous-type mice at embryonic day 13.5 (E13.5) with a dose of 50 mg/kg. Two hours post injection, brains were dissected for proliferation assays. For migration assay, 7 days post injection, brains were harvested for subsequence assays (Supplementary Figure 4A). Detailed information about proliferation and migration assays are provided in Supplementary methods.

### Cortical development evaluation

Brains from P0 wild-type (SLC39A8-p.393A) and knock-in (SLC39A8-p.393T) mice were fixed with 4% paraformaldehyde (PFA), dehydrated by 30% sucrose, and then embedded into Tissue-Tek O.C.T. compound (Cat. No: 4583, SAKURA) at −80 °C. Coronal sections with a thickness of 10 μm were prepared by freezing microtome (Thermo Scientific, CryoStar NX50, America) at −22 °C. Coronal sections from the same cortical regions of wild-type and knock-in mice were selected to perform immunohistochemical staining. Four cortical layer-specific markers (TBR1, CTIP2, SOX2, SATB2) were used as previous paper to label the primary somatosensory cortex [31, 32]. The primary antibodies used were rabbit anti-TBR1 (Cat. No: ab31940, Abcam), rat anti-CTIP2 (Cat. No: ab18465, Abcam), mouse anti-SOX2 (Cat. No: GB14149, Servicebio), rabbit anti-SATB2 (Cat. No: GB111449, Servicebio). The secondary antibodies were anti-rabbit 488 (Cat. No: GB25303, Servicebio), anti-rat cy3 (Cat. No: GB21302, Servicebio), anti-mouse 488 (Cat. No: GB25301, Servicebio), and anti-rabbit cy3 (Cat. No: GB21303, Servicebio). The positive cell*s* (TBR1^+^, CTIP2^+^, SOX2^+^, SATB2^+^) and DAPI^+^ were counted by using Image J software (https://imagej.nih.gov/ij/) and the mouse genotypes were validated by Sanger sequencing. Coronal sections were photographed by laser scanning confocal microscope (FV1000, OLYMPUS) with 40× under the oil lens. A total of 48 photographs from 6 mice (knock-in: *n* = 3; wild-type: *n* = 3) were used for evaluating the cortical development whether affected by SLC39A8-p.393T.

### Behavioral and cognitive analysis

All behavioral experiments were performed using adult male (ages: 8–12 weeks; weights: 18–26 g) wild-type (SLC39A8-p.393A) and knock-in (SLC39A8-p.393T) mice. All tested mice were placed into behavioral test house to habituate the environment at least for 1 h before behavioral tests. Detailed information about behavioral and cognitive analysis is provided in Supplementary methods.

### Analysis of dendritic spine density

Golgi staining is usually used to classify and quantify dendritic spines [33, 34]. Golgi staining was performed using the FD Rapid GolgiStainTM Kit (Cat. No: PK401A, FD NeuroTechnologies), following the manufacturer’s instructions. Detailed information about dendritic spine density analysis is provided in Supplementary methods.

### Electrophysiological recording on acute slices from hippocampus

Acute hippocampal slices were prepared from P18-21 wild-type (SLC39A8-p.393A) and knock-in (SLC39A8-p.393T) mice as previously described [35]. Briefly, mice were anesthetized with isoflurane and 300 μm transverse hippocampal slices were cut using a Leica VT1200 Vibratome in chilled high sucrose cutting solution, which is consisted of 2.5 mM KCl, 0.5 mM CaCl_2_, 7 mM MgCl_2_, 1.25 mM NaH_2_PO_4_, 25 mM NaHCO_3_, 7 mM D-glucose, 210 mM Sucrose, and 1.3 mM Ascorbic acid. The 300-μm-thick slices were then incubated for 30 min at 34 °C in artificial cerebrospinal fluid (ACSF) containing the following component: 119 mM NaCl, 2.5 mM KCl, 26.2 mM NaHCO_3_, 1 mM NaH_2_PO_4_, and 11 mM D-glucose. Then 2.5 mM CaCl_2_ and 1.3 mM MgSO_4_ were added into the ACSF, the pH of acute slices was maintained at ACSF with 95% O_2_/5% CO_2_. After 30 min incubation, the acute slices were placed at room temperature for 30–60 min and then recorded at room temperature.

For recording, individual slices were transferred to a chamber mounted in an up-right microscope (BX51WI, Olympus) and perfused with ASCF (2.5 mL/min). Pyramidal neurons were identified by morphology in hippocampal CA1 region. The internal recording solution contained: 135 mM CsMeSO_4_, 8 mM NaCl, 10 mM HEPES, 0.3 mM EGTA, 5 mM QX314-Cl, 4 mM Mg-ATP, 0.3 mM Na-GTP, and 0.1 mM spermine. The osmolarity was adjusted to 290–295 mOsm, and pH was buffered at 7.3–7.4. Miniature excitatory postsynaptic currents (mEPSCs) were recorded at −70 mV with 0.5 μM tetrodotoxin (TTX) in the ACSF. Whole-cell electrophysiological signals were collected with a Multiclamp 700B amplifier (Axon Instruments), filtered at 2 kHz, and digitized at 10 kHz. Custom software (IGOR Pro) was used to analyze the data offline. Statistical analyses were compared to respective controls with Mann–Whitney U test with Bonferonni correction.

## Results

### SLC39A8-p.393T did not affect global growth of the knock-in mice

**Fig. 1:**
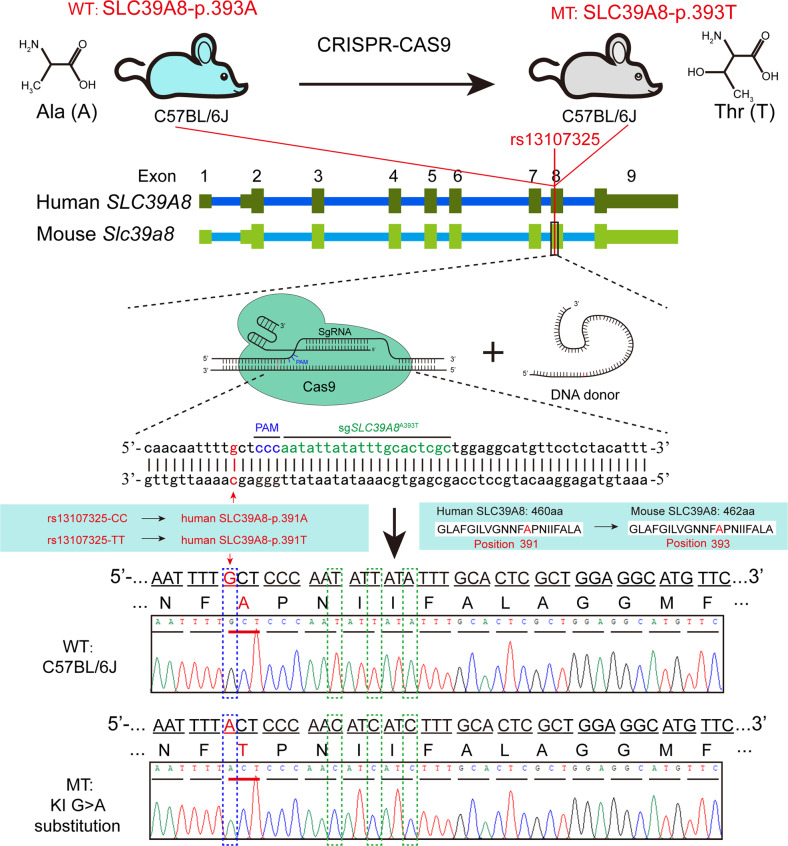
Generation of the SLC39A8-p.393T knock-in mouse model. CRISPR-Cas9-mediated genome editing was used to construct the SLC39A8-p.393T knock-in mouse model. Human and mouse *SLC39A8* gene showed high-degree sequence and structure similarities. The missense SNP rs13107325 is located in the eighth exon of *SLC39A8* and encodes p.Ala391T amino acid, which corresponds to mouse SLC39A8-p.393. Sanger sequencing showed that SLC39A8-p.393T knock-in mouse model was successfully generated. The position of SLC39A8-p.393T (corresponds to human rs13107325) was marked by blue dashed box. WT: wild-type mice (SLC39A8-p.393A); MT: knock-in mice (SLC39A8-p.393T).

**Fig. 2:**
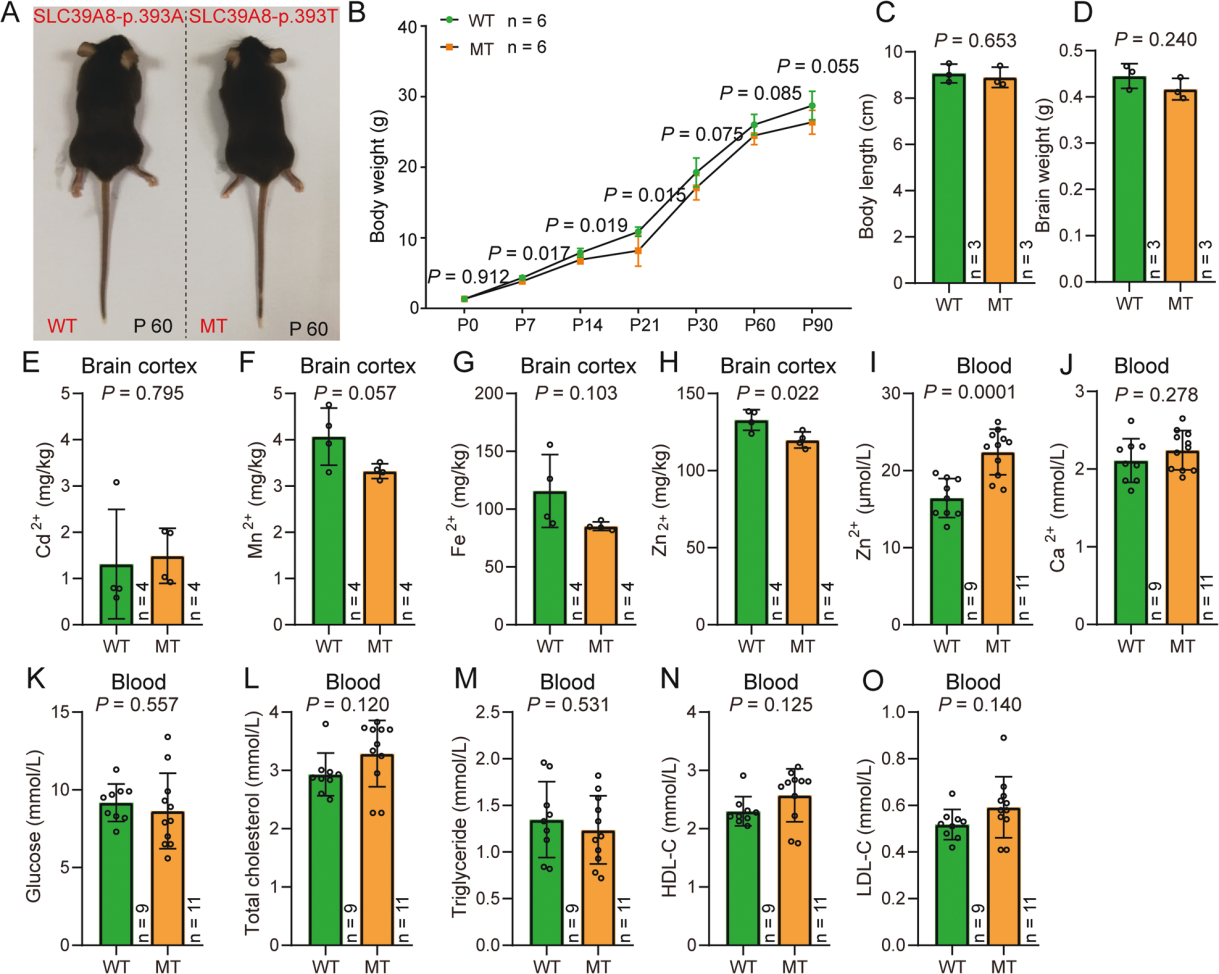
Impact of SLC39A8-p.393T knock-in on global growth and ions concentration. **A**–**C** SLC39A8-p.393T knock-in did not affect body length and weight. **D** SLC39A8-p.393T knock-in did not affect brain weight. **E**–**G** SLC39A8-p.393T knock-in did not affect cadmium, manganese and iron concentration in brain tissues. **H** SLC39A8-p.393T knock-in resulted in down-regulation of zinc concentration in brain tissues. **I** SLC39A8-p.393T knock-in resulted in up-regulation of zinc concentration in blood. **J**–**O** SLC39A8-p.393T knock-in did not affect calcium concentration, glucose, cholesterol, triglyceride, HDL-C and LDL-C. Two-tailed Student’s *t***-**test was used for statistical test. Data represent mean ± SD, *n* = 6 for (**B**), *n* = 3 for (**C** and **D**), *n* = 4 for (**E**–**H**), *n* (WT) = 9 and *n* (MT) = 11 for (**J**–**O**).

By using CRISPR-Cas9-mediated genome editing, we successfully obtained the SLC39A8-p.393T knock-in mice (Fig. 1). Compared with wild-type mice (which carried alanine at p.393), the amino acid of SLC39A8 at p.393 was changed to threonine (SLC39A8-p.393T) in knock-in mice (Fig. 1). The SLC39A8-p.393T knock-in mice thus can model the effect of risk allele of rs13107325 observed in human. We firstly evaluated the effect of SLC39A8-p.393T on body weight and length, and found that the knock-in mice did not show significant differences in body weight and length compared with wild-types at P60 stage (Fig. 2A–C), though significant body weight differences were observed at P14 to P30 stages. Further analysis indicated there was no significant difference in brain weight of the knock-in and wild-type mice (Fig. 2D). These results indicate that SLC39A8-p.393T did not affect global growth (including body and brain weight) of the knock-in mice.

### SLC39A8-p.393T alters zinc concentration in brain and blood

SLC39A8 is a transporter for metal ions cadmium, zinc, manganese, and iron. To investigate if SLC39A8-p.393T affects transport of these metal ions, we measured the concentration of cadmium, zinc, manganese, and iron in brains and blood of the knock-in and wild-type mice. Cadmium, manganese, and iron did not show significant changes in brains of the knock-in and wild-type mice (Fig. 2E–G). However, concentration of zinc was significantly down-regulated in brains of the knock-in mice compared with wild-types (Fig. 2H), indicating that SLC39A8-p.393T impaired zinc uptake ability in the mouse brain. We further measured zinc concentration in blood and found that zinc concentration in blood of the knock-in mice was significantly up-regulated compared with wild-types (Fig. 2I). This result indicates that SLC39A8-p.393T impairs the zinc transport ability and leads to accumulation and elevated zinc concentration in the blood of the knock-in mice.

Considering that previous GWASs also reported significant association between rs13107325 and circulating high-density lipoprotein cholesterol (HDL-C) [36, 37], we measured the concentration of glucose, total cholesterol, triglyceride, HDL-C, and low-density lipoprotein cholesterol (LDL-C) in blood of SLC39A8-p.393T knock-in and wild-type mice. None of these tested lipids showed difference in blood of SLC39A8-p.393T knock-in and wild-type mice (Fig. 2L–O). These data indicate that SLC39A8-p.393T regulates zinc transport in brains and blood of the knock-in mice.

### SLC39A8-p.393T did not affect proliferation and migration of neural stem cells

Previous studies have demonstrated the crucial roles of schizophrenia risk genes in regulating proliferation and migration of neural stem cells [38–40]. To explore if SLC39A8-p.393T knock-in mice exhibited abnormalities in proliferation and migration of NSCs in vivo, we performed EdU incorporation assays. EdU assays revealed no significant differences in proliferation and migration of NSCs in SLC39A8-p.393T knock-in and wild-type mice (Supplementary Figure 4), suggesting no obvious effect of SLC39A8-p.393T knock-in on proliferation and migration of NSCs.

### Subtle effect of SLC39A8-p.393T on cortical development

**Fig. 3:**
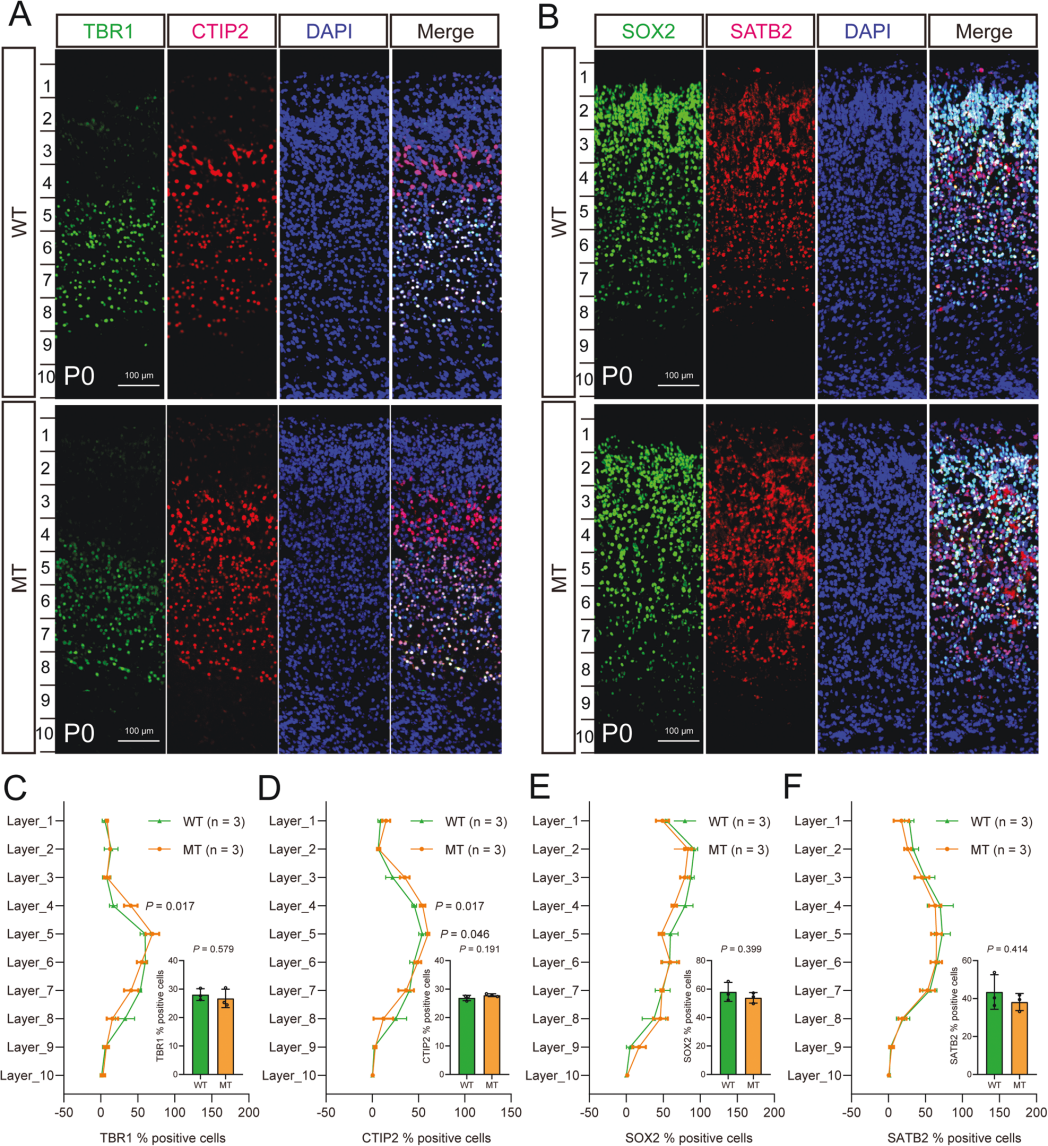
Subtle effect of SLC39A8-p.393T on cortical development. **A**, **B** Representative immunofluorescence images for four cortical layer-specific markers (TBR1, CTIP2, SOX2, SATB2) (P0 stage). **C**–**F** The subtle effect of SLC39A8-p.393T knock-in on cortical development. The statistical result of **A** was showed in **C**, **D**, the statistical result of **B** was showed in **E**, **F**. Two-tailed Student’s test was used for statistical test. Data represent mean ± SD, *n* = 3 for (**C**–**F**).

Although NSCs of the SLC39A8-p.393T knock-in mice did not exhibit abnormalities in proliferation and migration, whether cortical development was affected remains unclear. We thus investigated the effect of SLC39A8-p.393T knock-in on cortical development by using four cortical layer-specific markers (TBR1, CTIP2, SOX2, SATB2) [31, 32]. We found that the ratios of TBR1^+^/DAPI^+^, CTIP2^+^/DAPI^+^, SOX2^+^/DAPI^+^, and SATB2^+^/DAPI^+^ did not show significant differences between knock-in mice and wild-types (Fig. 3). However, when we further divided the cortex into 10 layers (as described in Moon’s study [41]), we found that the ratio of CTIP2^+^/DAPI^+^ was significantly higher at layer 4 and 5 in knock-in mice compared with wild-types. In addition, we also found that the ratio of TBR1^+^/DAPI^+^ was significantly higher at layer 4 in knock-in mice compared with wild-types (Fig. 3 C, D). These results indicated the subtle effect of SLC39A8-p.393T on cortical development.

### Subtle effect of SLC39A8-p.393T on transcription regulation

**Fig. 4:**
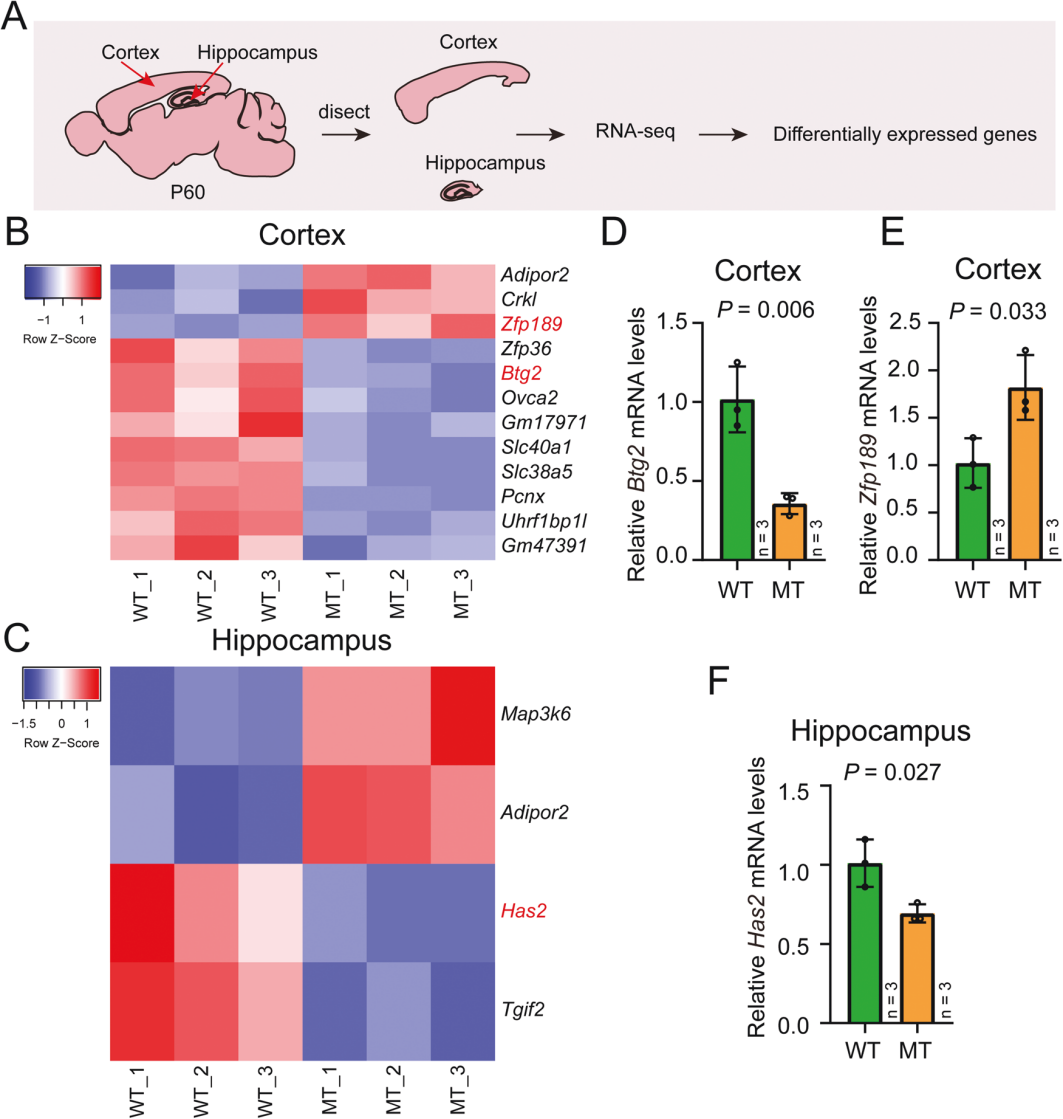
Impact of SLC39A8-p.393T knock-in on gene expression in the cortex and hippocampus. **A** Overview of transcriptome analysis. **B**, **C** DEGs identified in the cortex and hippocampus of SLC39A8-p.393T knock-in and wild-type mice, respectively. **D**–**F** qPCR validation of the RNA sequencing. Two-tailed Student’s *t***-**test was used for statistical test. Data represent mean ± SD, *n* = 3 for **D**–**F**.

We next investigated the effect of SLC39A8-p.393T knock-in on gene expression in mouse brain (from P60 mice). Cortical and hippocampal tissues of SLC39A8-p.393T knock-in and wild-type mice were dissected firstly, then RNA sequencing (RNA-seq) were conducted to detect the DEGs in SLC39A8-p.393T knock-in and wild-type mice (Fig. 4A). A total of 12 DGEs were identified in cortical tissues of SLC39A8-p.393T knock-in compared with wild-type mice. Among the DEGs, 9 were down-regulated and 3 were up-regulated (Fig. 4B). In hippocampus, only 4 DEGs (*Map3k6, Adipor2, Has2*, and *Tgif2*) were identified (Fig. 4C). Real-time quantitative PCR (by randomly selecting 3 genes, *Btg2, Zfp189*, and *Has2*) validated the RNA-seq results (Fig. 4D–F). Collectively, these results indicate subtle effect of SLC39A8-p.393T on transcription regulation in cortical tissues and hippocampus.

### No significant behavioral and cognitive abnormalities in SLC39A8-p.393T knock-in mice compared with wild-types

To investigate if SLC39A8-p.393T affects behavioral and cognitive functions, we performed serial behavioral and cognitive tests, including open-field, light-dark transition, elevated plus maze, novel object recognition, Y-maze, 3-chambered social approach, tail suspension test. Only the total distance and distance in corner of knock-in mouse were significantly decreased compared with wild-types in the open-field test (Supplementary Figure 5B and D). Other tested behavioral and cognitive tests did not show abnormalities in SLC39A8-p.393T knock-in mice compared with wild-types (Supplementary Figures 5–7).

### Decreased dendritic spine density in SLC39A8-p.393T knock-in mice compared with wild-types

**Fig. 5:**
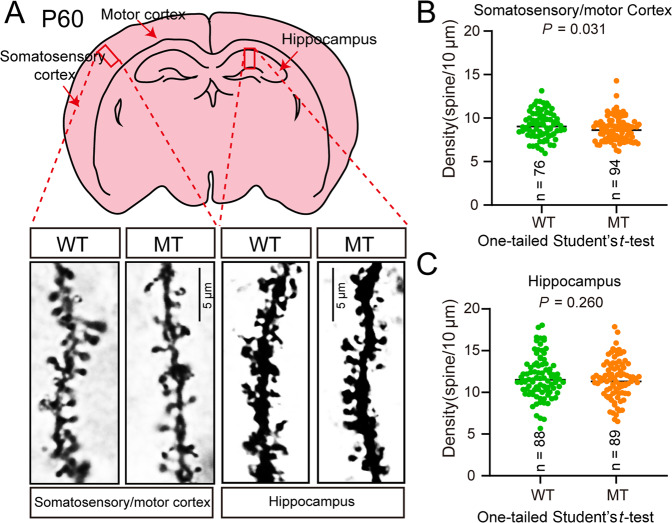
SLC39A8-p.393T knock-in affected dendritic spine density. **A** Overview of dendritic spine analysis. **B**, **C** Dendritic spine density in cortex was significantly decreased in SLC39A8-p.393T knock-in mice compared with wild-types. One-tailed Student’s *t***-**test was used for statistical test. Data represent mean ± SD, the number of neurons used for this quantitative analysis in WT and MT groups were *n* = 76 and *n* = 94 in cortex (**B**), *n* = 88 and *n* = 89 in hippocampus (**C**) (from 3 independent SLC39A8-p.393T knock-in mice and 3 SLC39A8-p.393A wild-type mice).

Our above results indicated that SLC39A8-p.393T did not affect global growth (including body and brain weight). Consistent with this, proliferation and migration of NSCs did not show significant differences in SLC39A8-p.393T knock-in mice compared with wild-types. In addition, SLC39A8-p.393T knock-in mice did not exhibit obvious behavioral and cognitive abnormalities, compared with wild-types. We thus focused on dendritic spine density, a featured characteristic that was frequently reported to be decreased in schizophrenia [28–30, 42]. Compared with wild-types, dendritic spine density of SLC39A8-p.393T knock-in mice was significantly decreased in cortex (*P* = 0.031, one-tailed Student’s *t* test) (Fig. 5B). This finding is interesting as previous studies have repeatedly reported decreased dendritic spine density in schizophrenia [28–30, 42, 43]. Of note, our finding is consistent with results observed in schizophrenia cases (i.e., decreased dendritic spine density) [29, 30, 42], indicating that SLC39A8-p.393T could regulate dendritic spine morphogenesis. This result indicates that SLC39A8-p.393T knock-in resulted in decreased dendritic spine density, thus mimicked the dendritic spine pathology observed in schizophrenia. This result also suggests that rs13107325 might confer schizophrenia risk by regulating dendritic spine density, a featured characteristic that was frequently reported to be decreased in schizophrenia. However, dendritic spine density in hippocampus did not show difference in SLC39A8-p.393T knock-in mice compared with wild-types (*P* = 0.260, one-tailed Student’s *t* test) (Fig. 5C), suggesting that the effect of SLC39A8-p.393T on dendritic spine density is brain region-dependent. In summary, our knock-in mouse model supports the functionality of rs13107325 and indicates that this missense variant might confer schizophrenia risk by regulating zinc transport and dendritic spine density.

### SLC39A8-p.393T knock-in mice did not show abnormal synaptic transmission

**Fig. 6:**
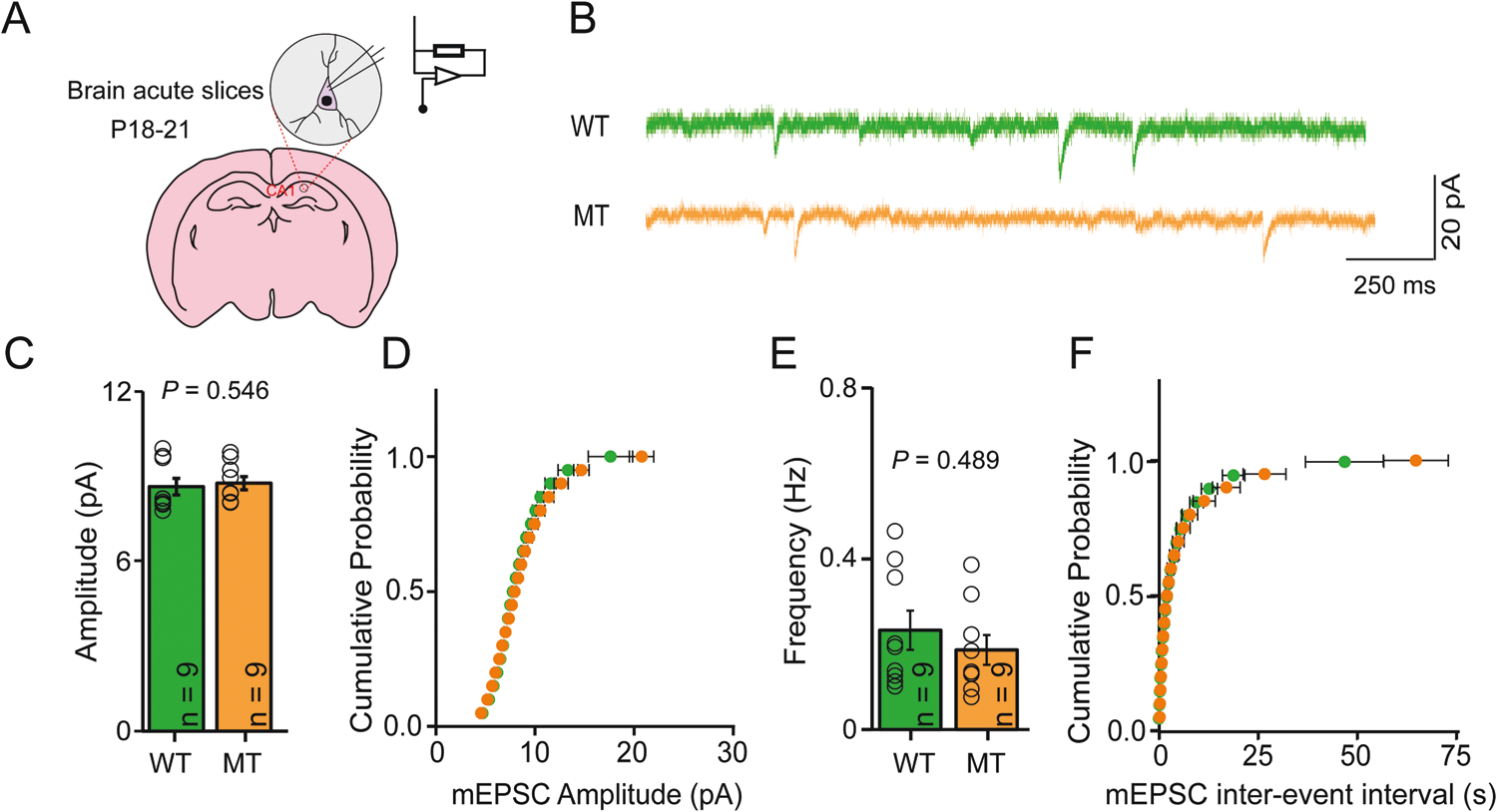
Both the amplitude and the frequency of mEPSCs showed no significant difference between SLC39A8-p.393T knock-in mice and wild-types. **A** Overview of electrophysiological recording on acute slices from hippocampus. **B** Representative traces of mEPSC in wild-types (green) and SLC39A8-p.393T knock-in mice (orange) CA1 neurons. (Scale bars (20 pA and 250 ms) for single representative mEPSC traces). **C** The mEPSC amplitude did not change significantly in SLC39A8-p.393T knock-in neurons compared with wild-types (*P* = 0.546) (WT: 8.66 ± 0.29 pA, MT: 8.78 ± 0.24 pA). **D** Cumulative distribution functions of mEPSC amplitude showed no irregularities between SLC39A8-p.393T knock-in mice (green) and wild-types (black). **E** There was no significant change in mEPSCs frequency between SLC39A8-p.393T knock-in neurons and wild-types (*P* = 0.489) (WT: 0.23 ± 0.05 Hz, MT: 0.19 ± 0.03 Hz). **F** Cumulative distribution functions of mEPSC frequency showed no irregularities between SLC39A8-p.393T knock-in mice (green) and wild-types (black). Mann–Whitney U test with Bonferonni correction was used for statistical test, data represent mean ± SD, *n* = 9 for **C**–**F**.

We investigated the effect of SLC39A8-p.393T on synaptic transmission by evaluating the properties of mEPSCs. We recorded mEPSCs from hippocampal CA1 pyramidal neurons by blocking sodium channels with 0.5 μM tetrodotoxin (TTX) on acute slices at postnatal days 18–21 mice. We found that neither the mEPSCs amplitude nor frequency of SLC39A8-p.393T knock-in mice showed any difference from that of wild-types (Fig. 6C–F), suggesting that the SLC39A8-p.393T has no effect on synaptic transmission regulation.

## Discussion

Among the schizophrenia risk variants identified by GWASs, the missense SNP rs13107325 is one of the most probable and promising causal variants [5, 16]. Statistical fine-mapping [5], conservation analysis [21], functional annotation [22–24], physicochemical property differences of the amino acid encodes by different alleles of rs13107325, and functional characterizations supported the functionality and causality of this missense variant [25, 26]. Though lines of evidence support the potential pathogenicity of rs13107325 and several studies have explored the biological effect of this non-synonymous variant in cell or animal models, whether rs13107325 is a pathogenic variant for schizophrenia remains unknown. To elucidate the role of rs13107325 in schizophrenia, we generated a knock-in mouse model by introducing a point mutation at SLC39A8-p.393T, which corresponds to human rs13107325 (SLC39A8-p.Ala393Thr). Considering the pleiotropic effect of rs13107325 [5, 17, 18, 24, 36], we assessed many rs13107325-associated phenotypes or traits using this knock-in mouse model, including body and brain weight, concentrations of metal ions (including cadmium, zinc, manganese, and iron) transported by SLC39A8, lipids, gene expression, behavioral and cognitive tests, proliferation and migration of NSCs, cortical development, dendritic spine density as well as synaptic transmission.

Point mutation mouse model provides a pivotal opportunity to decipher the biological effects of missense risk variants in disease susceptibility and complex traits [44–47]. By evaluating the phenotypes and traits of SLC39A8-p.393T knock-in mice, we observed several interesting results. The first important finding of this study is that we found that SLC39A8-p.393T affected zinc concentration in brain and blood of the knock-in mice, validating the functionality of rs13107325. The second interesting finding is the subtle effect of SLC39A8-p.393T on cortical development, though the underlying mechanism remains unclear. The third intriguing finding is the decreased dendritic spine density observed in SLC39A8-p.393T knock-in mice. So far, the pathology of schizophrenia remains largely unknown. Nevertheless, accumulating evidence suggests that dysfunction of dendritic spines plays a crucial role in schizophrenia [28–30, 42, 43, 48, 49]. First, reduced dendritic spine density was frequently reported in schizophrenia [30, 42, 43]. Second, many schizophrenia risk genes have been reported to be involved in dendritic spine morphogenesis [50–53]. Third, a recent study by Radhakrishnan et al. demonstrated that decreased synaptic spine density is an intrinsic characteristic in schizophrenia [54]. These studies indicate that dysregulation of dendritic spines may have a critical role in schizophrenia. Consistent with these studies, we observed decreased dendritic spine density in cortex in SLC39A8-p.393T knock-in compared with wild-type mice. It should be noted that the synaptic transmission of pyramidal neurons in hippocampus showed no differences in wild-type and SLC39A8-p.393T knock-in mice (Fig. 6C–F). More work is needed to investigate the role of rs13107325 in schizophrenia pathogenesis. In summary, we successfully linked the risk missense variant rs13107325 to a featured characteristic of schizophrenia by using the SLC39A8-p.393T knock-in mouse model. Our study clearly shows the importance of rs13107325 in dendritic spine morphogenesis, providing a reasonable biological explanation between rs13107325 and schizophrenia.

rs13107325 is a highly pleiotropic missense variant that showed strong association with many complex human traits and diseases, including schizophrenia [5, 15], blood lipids [36, 55], steatohepatitis [56], intelligence [57], Crohn’s disease [58], severe idiopathic scoliosis [59], brain structure [60–62], and others. The risk allele of rs13107325 only appears in European (8%) and American (5%) populations, but not in Asian and African populations (Supplementary Figure 2). Our previous study showed that the risk allele of rs13107325 might experience recent positive selection in Europeans, indicating this variant may act as a double-edged sword. The evolutionary selection shaped the frequency distribution of the risk allele of rs13107325 in world populations [21].

We did not detect differences in blood lipids, behaviors, and cognition between SLC39A8-p.393T knock-in and wild-type mice (Fig. 2). First, A possible explanation is the small number of the SLC39A8-p.393T knock-in and wild-type mice used in this study. Due to the huge time cost and expenditure, obtaining a large number of SLC39A8-p.393T knock-in mice is laborious and challenging. Thus, only limited SLC39A8-p.393T knock-in and wild-type mice were used for blood lipids detection, which may limit the statistical power of our study (considering the small effect of risk variants identified by GWASs). Second, the species differences (including genome, morphology, physiology, etc.) between human and mouse may also lead to the results observed in this study. Third, considering the polygenicity nature of complex traits and diseases, it is likely that many variants (each has a small effect size) act synergistically to affect the complex traits. Nevertheless, we only considered a single variant in this study. Fourth, the advanced cognition such as associative memory, logical thinking, intelligence, is difficult to evaluate in mice. Fifth, we explored *SLC39A8* expression in single cells from the human brain (CoDEx Viewer: http://solo.bmap.ucla.edu/shiny/webapp/, UCSC cell browser: https://cells.ucsc.edu/?ds=autism, and Transcriptomics Explorer of Allen brain map: https://celltypes.brain-map.org/rnaseq/human_ctx_smart-seq?selectedVisualization=Heatmap&colorByFeature=Cell+Type&colorByFeatureValue=GAD1), and found that *SLC39A8* is not widely expressed in different cell types of the human brain (Supplementary Figures 8, 9). Thus, more work is needed to further investigate how this missense variant confer risk of schizophrenia. Finally, the interaction between genetic and environmental factors also plays a pivotal role in regulating complex traits. Further work which considering both genetic and environmental factors are necessary to illuminate the role and mechanism of this missense variant in schizophrenia.

rs13107325 lies in the eighth exon of *SLC39A8*, which encodes ZIP8, a member of the solute-carrier-39 (SLC39) metal-transporter family. SLC39A8 is widely expressed in diverse human tissues (data were from the Human Protein Atlas, https://www.proteinatlas.org/) (Supplementary Figure 10–11), with relatively high expression in lung, kidney, endometrium, placenta. This wide expression pattern suggests the important role of SLC39A8 in diverse human tissues. Consistent with this, multiple studies have demonstrated the pivotal physiological function of SLC39A8. For example, Besecker et al. revealed the important role of SLC39A8 in zinc-mediated cytoprotection in lung epithelia [63]. Studies by Zhang et al. and Fujishiro et al. showed that SLC39A8 has a crucial role in intracellular cadmium accumulation and cell toxicity [25, 64]. Liu et al. found that SLC39A8 regulates host defense by inhibiting NF-kB [65]. Ding et al. also found that SLC39A8 mediates the degradation of extracellular matrix via NF-κB signaling pathway [66]. Kim et al. demonstrated the critical role of SLC39A8 in osteoarthritis [67]. Of note, the study by Galvez-Peralta et al. indicated that SLC39A8 has an indispensable role for both organogenesis and hematopoiesis [68]. In addition to these findings, human genetic studies also showed the vital role of SLC39A8. Park et al. reported that SLC39A8 mutation was associated with congenital disorder of glycosylation by impairing β-1,4-galactosyltransferase and manganese uptake [69, 70]. Interestingly, a recent study by Tseng et al. showed that the human SLC39A8-p.391T missense variant (corresponds to the risk allele of rs13107325) reduced zinc transport (into the cell) in cell models [71], which was consistent with our observations in this study (i.e., zinc concentration was dysregulated in SLC39A8-p.391T knock-in mice compared with wild-types). Zn^2+^ has a critical role in the brain, including regulating synaptic plasticity through microtubule stability [72], CTTNBP2 condensates [73], and BDNF signaling [74]. Consistent with its important roles in the brain, Zinc concentration has been reported to be associated with several neuropsychiatric disorders [75], including schizophrenia [76, 77] and Alzheimer’s disease [78]. Zn^2+^ is mainly distributed in the central nervous system (CNS) and stored in synaptic vesicles at several glutamatergic nerve terminals, and released upon its neuronal activity [79, 80]. The dopamine hypothesis of schizophrenia suggests that the dopamine D1 receptor neurotransmission in the prefrontal cortex of schizophrenia is hypo-functional [81]. The dopamine transporter (DAT) can terminate synaptic transmission by reuptake of extracellular dopamine, and the action of Zn^2+^ on DAT had dual directions (stimulation or inhibition) depends on intracellular Na^+^ concentration [82]. These lines of evidence indicated that the abnormal Zn^2+^ levels might participate in pathogenesis of neuropsychiatric disorders. Considering the important roles (including neurogenesis and synaptic transmission) of zinc in the brain [75, 83, 84], it is possible that rs13107325 might confer schizophrenia risk through affecting zinc transport.

Another amazing finding of this study is that only limited genes showed differential expression in cortical and hippocampal tissues of the SLC39A8-p.393T knock-in and wild-type mice. Only 12 and 4 DEGs were identified in cortical and hippocampal tissues, respectively, indicating the weak regulation effect of the SLC39A8-p.393T on gene expression in the brain. We also examined if the gene-editing process (i.e., SLC39A8-p.393T knock-in) affects *Slc39a8* mRNA expression in mouse brain. We firstly checked if the gene-editing process affects *SLC39A8* transcription using RNA sequencing data. We found that the gene-editing process did not affect *Slc39a8* mRNA expression in the brain (Supplementary Figure 12A, B). Moreover, we also collected the cortical and hippocampal tissues from SLC39A8-p.393T knock-in mice and wild-types (5-month-old), and conducted quantitative PCR. Again, we found that *Slc39a8* mRNA level did not show difference between SLC39A8-p.393T knock-in mice and wild-types (Supplementary Figure 12C, D). These findings suggest that rs13107325 may exerts its main effect by affecting SLC39A8 function (but not through regulating gene expression). Comparison of DEGs in cortex and hippocampus identified *Adipor2* as the unique gene that up-regulated in both cortex and hippocampus. *Adipor2* encodes adiponectin Receptor Protein 2 (ADIPOR2), which is mainly expressed in liver and promotes glucose uptake and fatty acid oxidation by binding to adiponectin [85]. Considering the significant association between rs13107325 and blood lipids [36, 55], more work is needed to explore if rs13107325 regulates the level of blood lipids by modulating *Adipor2* expression. Other interesting genes include *Zfp189* (which encodes zinc finger protein and is associated with stress resilience [86]), *Zfp36* (a gene plays a role in insulin resistant and fat liver [87]), and *Btg2* (encodes BTG anti-proliferation factor 2, a protein that is involved in cell cycle regulation, growth arrest and differentiation of the neuronal precursors, and neurite outgrowth [88–91]). Finally, we conducted qPCR to explore the expression of *Slc39a8* during development. Whole brain tissues were collected at E13.5, E15.5, E17.5, P0, P7, P14, P30, and P60 stages, and qPCR were performed. We found that the *Slc39a8* has the highest expression at P7. *Slc39a8* expression is relatively low and stable at other developmental stages (Supplementary Figure 12 E). These results suggest the important role of SLC39A8 in the central nervous system.

In summary, we generated the SLC39A8-p.393T knock-in mouse model and we extensively characterized the phenotypes and traits of this knock-in mouse model. Although we did not detect significant differences in many traits (associated with rs13107325 in humans) in SLC39A8-p.393T knock-in and wild-type mice, we validated the functionality of rs13107325 and our study indicate that rs13107325 may confer schizophrenia risk by affecting dendritic spines.

## Supplementary information

Supplementary_Material

## References

[1] O’Donovan MC, Craddock N, Norton N, Williams H, Peirce T, Moskvina V (2008). Identification of loci associated with schizophrenia by genome-wide association and follow-up. Nat Genet.

[2] Yue WH, Wang HF, Sun LD, Tang FL, Liu ZH, Zhang HX (2011). Genome-wide association study identifies a susceptibility locus for schizophrenia in Han Chinese at 11p11.2. Nat Genet.

[3] Shi J, Levinson DF, Duan J, Sanders AR, Zheng Y, Pe’er I (2009). Common variants on chromosome 6p22.1 are associated with schizophrenia. Nature.

[4] Shi Y, Li Z, Xu Q, Wang T, Li T, Shen J (2011). Common variants on 8p12 and 1q24.2 confer risk of schizophrenia. Nat Genet.

[5] Schizophrenia Working Group of the Psychiatric Genomics Consortium. (2014). Biological insights from 108 schizophrenia-associated genetic loci. Nature.

[6] Lam M, Chen CY, Li Z, Martin AR, Bryois J, Ma X (2019). Comparative genetic architectures of schizophrenia in East Asian and European populations. Nat Genet.

[7] Cousin MA, Creighton BA, Breau KA, Spillmann RC, Torti E, Dontu S (2021). Pathogenic SPTBN1 variants cause an autosomal dominant neurodevelopmental syndrome. Nat Genet.

[8] Asgari S, Luo Y, Akbari A, Belbin GM, Li X, Harris DN (2020). A positively selected FBN1 missense variant reduces height in Peruvian individuals. Nature.

[9] Yang SK, Hong M, Baek J, Choi H, Zhao W, Jung Y (2014). A common missense variant in NUDT15 confers susceptibility to thiopurine-induced leukopenia. Nat Genet.

[10] Huang L, Zhang H, Cheng CY, Wen F, Tam PO, Zhao P (2016). A missense variant in FGD6 confers increased risk of polypoidal choroidal vasculopathy. Nat Genet.

[11] Hampe J, Franke A, Rosenstiel P, Till A, Teuber M, Huse K (2007). A genome-wide association scan of nonsynonymous SNPs identifies a susceptibility variant for Crohn disease in ATG16L1. Nat Genet.

[12] Weile J, Kishore N, Sun S, Maaieh R, Verby M, Li R (2021). Shifting landscapes of human MTHFR missense-variant effects. Am J Hum Genet.

[13] Van Hout CV, Tachmazidou I, Backman JD, Hoffman JD, Liu D, Pandey AK (2021). Exome sequencing and characterization of 49,960 individuals in the UK Biobank. Nature.

[14] Vannucchi AM, Verstovsek S, Guglielmelli P, Griesshammer M, Burn TC, Naim A (2017). Ruxolitinib reduces JAK2 p.V617F allele burden in patients with polycythemia vera enrolled in the RESPONSE study. Ann Hematol.

[15] Carrera N, Arrojo M, Sanjuan J, Ramos-Rios R, Paz E, Suarez-Rama JJ (2012). Association study of nonsynonymous single nucleotide polymorphisms in schizophrenia. Biol Psychiatry.

[16] Pardinas AF, Holmans P, Pocklington AJ, Escott-Price V, Ripke S, Carrera N (2018). Common schizophrenia alleles are enriched in mutation-intolerant genes and in regions under strong background selection. Nat Genet.

[17] Pickrell JK, Berisa T, Liu JZ, Segurel L, Tung JY, Hinds DA (2016). Detection and interpretation of shared genetic influences on 42 human traits. Nat Genet.

[18] Costas J (2017). The highly pleiotropic gene SLC39A8 as an opportunity to gain insight into the molecular pathogenesis of schizophrenia. Am J Med Genet B Neuropsychiatr Genet.

[19] He L, Girijashanker K, Dalton TP, Reed J, Li H, Soleimani M (2006). ZIP8, member of the solute-carrier-39 (SLC39) metal-transporter family: characterization of transporter properties. Mol Pharm.

[20] Wang CY, Jenkitkasemwong S, Duarte S, Sparkman BK, Shawki A, Mackenzie B (2012). ZIP8 is an iron and zinc transporter whose cell-surface expression is up-regulated by cellular iron loading. J Biol Chem.

[21] Li M, Wu DD, Yao YG, Huo YX, Liu JW, Su B (2016). Recent positive selection drives the expansion of a schizophrenia risk nonsynonymous variant at SLC39A8 in Europeans. Schizophr Bull.

[22] Kircher M, Witten DM, Jain P, O’Roak BJ, Cooper GM, Shendure J (2014). A general framework for estimating the relative pathogenicity of human genetic variants. Nat Genet.

[23] Vogelezang S, Bradfield JP, Ahluwalia TS, Curtin JA, Lakka TA, Grarup N (2020). Novel loci for childhood body mass index and shared heritability with adult cardiometabolic traits. PLoS Genet.

[24] Sanchez-Roige S, Palmer AA, Fontanillas P, Elson SL, Adams MJ, Howard DM (2018). Genome-wide association study meta-analysis of the alcohol use disorders identification test (AUDIT) in two population-based cohorts. Am J Psychiatry.

[25] Zhang R, Witkowska K, Afonso Guerra-Assuncao J, Ren M, Ng FL, Mauro C (2016). A blood pressure-associated variant of the SLC39A8 gene influences cellular cadmium accumulation and toxicity. Hum Mol Genet.

[26] Nakata T, Creasey EA, Kadoki M, Lin H, Selig MK, Yao J (2020). A missense variant in SLC39A8 confers risk for Crohn’s disease by disrupting manganese homeostasis and intestinal barrier integrity. Proc Natl Acad Sci USA.

[27] Sunuwar L, Frkatović A, Sharapov S, Wang Q, Neu HM, Wu X (2020). Pleiotropic ZIP8 A391T implicates abnormal manganese homeostasis in complex human disease. JCI Insight.

[28] Penzes P, Cahill ME, Jones KA, VanLeeuwen JE, Woolfrey KM (2011). Dendritic spine pathology in neuropsychiatric disorders. Nat Neurosci.

[29] Glausier JR, Lewis DA (2013). Dendritic spine pathology in schizophrenia. Neuroscience.

[30] Konopaske GT, Lange N, Coyle JT, Benes FM (2014). Prefrontal cortical dendritic spine pathology in schizophrenia and bipolar disorder. JAMA Psychiatry.

[31] Hisaoka T, Nakamura Y, Senba E, Morikawa Y (2010). The forkhead transcription factors, Foxp1 and Foxp2, identify different subpopulations of projection neurons in the mouse cerebral cortex. Neuroscience.

[32] Miao N, Bian S, Lee T, Mubarak T, Huang S, Wen Z (2018). Opposite roles of Wnt7a and Sfrp1 in modulating proper development of neural progenitors in the mouse cerebral cortex. Front Mol Neurosci.

[33] Risher WC, Ustunkaya T, Singh Alvarado J, Eroglu C (2014). Rapid Golgi analysis method for efficient and unbiased classification of dendritic spines. PLoS One.

[34] Zaqout S, Kaindl AM (2016). Golgi-Cox staining step by step. Front Neuroanat.

[35] Sheng N, Bemben MA, Díaz-Alonso J, Tao W, Shi YS, Nicoll RA (2018). LTP requires postsynaptic PDZ-domain interactions with glutamate receptor/auxiliary protein complexes. Proc Natl Acad Sci USA.

[36] Teslovich TM, Musunuru K, Smith AV, Edmondson AC, Stylianou IM, Koseki M (2010). Biological, clinical and population relevance of 95 loci for blood lipids. Nature.

[37] Waterworth DM, Ricketts SL, Song K, Chen L, Zhao JH, Ripatti S (2010). Genetic variants influencing circulating lipid levels and risk of coronary artery disease. Arterioscler Thromb Vasc Biol.

[38] Mao Y, Ge X, Frank CL, Madison JM, Koehler AN, Doud MK (2009). Disrupted in schizophrenia 1 regulates neuronal progenitor proliferation via modulation of GSK3beta/beta-catenin signaling. Cell.

[39] Ishizuka K, Kamiya A, Oh EC, Kanki H, Seshadri S, Robinson JF (2011). DISC1-dependent switch from progenitor proliferation to migration in the developing cortex. Nature.

[40] Senturk A, Pfennig S, Weiss A, Burk K, Acker-Palmer A (2011). Ephrin Bs are essential components of the Reelin pathway to regulate neuronal migration. Nature.

[41] Moon UY, Park JY, Park R, Cho JY, Hughes LJ, McKenna J (2015). Impaired Reelin-Dab1 signaling contributes to neuronal migration deficits of tuberous sclerosis complex. Cell Rep.

[42] Glantz LA, Lewis DA (2000). Decreased dendritic spine density on prefrontal cortical pyramidal neurons in schizophrenia. Arch Gen Psychiatry.

[43] Moyer CE, Shelton MA, Sweet RA (2015). Dendritic spine alterations in schizophrenia. Neurosci Lett.

[44] Yamashita M, Kuehn HS, Okuyama K, Okada S, Inoue Y, Mitsuiki N (2021). A variant in human AIOLOS impairs adaptive immunity by interfering with IKAROS. Nat Immunol.

[45] El Ghaleb Y, Schneeberger PE, Fernandez-Quintero ML, Geisler SM, Pelizzari S, Polstra AM (2021). CACNA1I gain-of-function mutations differentially affect channel gating and cause neurodevelopmental disorders. Brain.

[46] Prieto M, Folci A, Poupon G, Schiavi S, Buzzelli V, Pronot M (2021). Missense mutation of Fmr1 results in impaired AMPAR-mediated plasticity and socio-cognitive deficits in mice. Nat Commun.

[47] Murthy A, Li Y, Peng I, Reichelt M, Katakam AK, Noubade R (2014). A Crohn’s disease variant in Atg16l1 enhances its degradation by caspase 3. Nature.

[48] Bennett MR (2011). Schizophrenia: susceptibility genes, dendritic-spine pathology and gray matter loss. Prog Neurobiol.

[49] MacDonald ML, Alhassan J, Newman JT, Richard M, Gu H, Kelly RM (2017). Selective loss of smaller spines in schizophrenia. Am J Psychiatry.

[50] Li Y, Ma C, Li W, Yang Y, Li X, Liu J (2021). A missense variant in NDUFA6 confers schizophrenia risk by affecting YY1 binding and NAGA expression. Mol Psychiatry.

[51] Deans PJM, Raval P, Sellers KJ, Gatford NJF, Halai S, Duarte RRR (2017). Psychosis risk candidate ZNF804A localizes to synapses and regulates neurite formation and dendritic spine structure. Biol Psychiatry.

[52] Sekar A, Bialas AR, de Rivera H, Davis A, Hammond TR, Kamitaki N (2016). Schizophrenia risk from complex variation of complement component 4. Nature.

[53] Yilmaz M, Yalcin E, Presumey J, Aw E, Ma M, Whelan CW (2021). Overexpression of schizophrenia susceptibility factor human complement C4A promotes excessive synaptic loss and behavioral changes in mice. Nat Neurosci.

[54] Radhakrishnan R, Skosnik PD, Ranganathan M, Naganawa M, Toyonaga T, Finnema S (2021). In vivo evidence of lower synaptic vesicle density in schizophrenia. Mol Psychiatry.

[55] Willer CJ, Schmidt EM, Sengupta S, Peloso GM, Gustafsson S, Kanoni S (2013). Discovery and refinement of loci associated with lipid levels. Nat Genet.

[56] Parisinos CA, Wilman HR, Thomas EL, Kelly M, Nicholls RC, McGonigle J (2020). Genome-wide and Mendelian randomisation studies of liver MRI yield insights into the pathogenesis of steatohepatitis. J Hepatol.

[57] Savage JE, Jansen PR, Stringer S, Watanabe K, Bryois J, de Leeuw CA (2018). Genome-wide association meta-analysis in 269,867 individuals identifies new genetic and functional links to intelligence. Nat Genet.

[58] Li D, Achkar JP, Haritunians T, Jacobs JP, Hui KY, D’Amato M (2016). A pleiotropic missense variant in SLC39A8 is associated with Crohn’s disease and human gut microbiome composition. Gastroenterology.

[59] Haller G, McCall K, Jenkitkasemwong S, Sadler B, Antunes L, Nikolov M (2018). A missense variant in SLC39A8 is associated with severe idiopathic scoliosis. Nat Commun.

[60] Hermann ER, Chambers E, Davis DN, Montgomery MR, Lin D, Chowanadisai W (2021). Brain magnetic resonance imaging phenome-wide association study with metal transporter gene SLC39A8. Front Genet.

[61] Pan X, Zhang M, Tian A, Chen L, Sun Z, Wang L (2022). Exploring the genetic correlation between obesity-related traits and regional brain volumes: Evidence from UK Biobank cohort. Neuroimage Clin.

[62] Luo Q, Chen Q, Wang W, Desrivières S, Quinlan EB, Jia T (2019). Association of a schizophrenia-risk nonsynonymous variant with putamen volume in adolescents: a voxelwise and genome-wide association study. JAMA Psychiatry.

[63] Besecker B, Bao S, Bohacova B, Papp A, Sadee W, Knoell DL (2008). The human zinc transporter SLC39A8 (Zip8) is critical in zinc-mediated cytoprotection in lung epithelia. Am J Physiol Lung Cell Mol Physiol.

[64] Fujishiro H, Okugaki S, Kubota K, Fujiyama T, Miyataka H, Himeno S (2009). The role of ZIP8 down-regulation in cadmium-resistant metallothionein-null cells. J Appl Toxicol.

[65] Liu MJ, Bao S, Galvez-Peralta M, Pyle CJ, Rudawsky AC, Pavlovicz RE (2013). ZIP8 regulates host defense through zinc-mediated inhibition of NF-kappaB. Cell Rep.

[66] Ding W, Ge Y, Sun H, Xu J, Gu H, Bian C (2021). ZIP8 mediates the extracellular matrix degradation of nucleus pulposus cells via NF-kappaB signaling pathway. Biochem Biophys Res Commun.

[67] Kim JH, Jeon J, Shin M, Won Y, Lee M, Kwak JS (2014). Regulation of the catabolic cascade in osteoarthritis by the zinc-ZIP8-MTF1 axis. Cell.

[68] Galvez-Peralta M, He L, Jorge-Nebert LF, Wang B, Miller ML, Eppert BL (2012). ZIP8 zinc transporter: indispensable role for both multiple-organ organogenesis and hematopoiesis in utero. PLoS ONE.

[69] Park JH, Hogrebe M, Gruneberg M, DuChesne I, von der Heiden AL, Reunert J (2015). SLC39A8 deficiency: a disorder of manganese transport and glycosylation. Am J Hum Genet.

[70] Park JH, Hogrebe M, Fobker M, Brackmann R, Fiedler B, Reunert J (2017). SLC39A8 deficiency: biochemical correction and major clinical improvement by manganese therapy. Genet Med.

[71] Tseng WC, Reinhart V, Lanz TA, Weber ML, Pang J, Le KXV (2021). Schizophrenia-associated SLC39A8 polymorphism is a loss-of-function allele altering glutamate receptor and innate immune signaling. Transl Psychiatry.

[72] Perrin L, Roudeau S, Carmona A, Domart F, Petersen JD, Bohic S (2017). Zinc and copper effects on stability of tubulin and actin networks in dendrites and spines of hippocampal neurons. ACS Chem Neurosci.

[73] Shih PY, Fang YL, Shankar S, Lee SP, Hu HT, Chen H (2022). Phase separation and zinc-induced transition modulate synaptic distribution and association of autism-linked CTTNBP2 and SHANK3. Nat Commun.

[74] Frazzini V, Granzotto A, Bomba M, Massetti N, Castelli V, d’Aurora M (2018). The pharmacological perturbation of brain zinc impairs BDNF-related signaling and the cognitive performances of young mice. Sci Rep.

[75] Kumar V, Kumar A, Singh K, Avasthi K, Kim JJ (2021). Neurobiology of zinc and its role in neurogenesis. Eur J Nutr.

[76] Cai L, Chen T, Yang J, Zhou K, Yan X, Chen W (2015). Serum trace element differences between Schizophrenia patients and controls in the Han Chinese population. Sci Rep.

[77] Saghazadeh A, Mahmoudi M, Shahrokhi S, Mojarrad M, Dastmardi M, Mirbeyk M (2020). Trace elements in schizophrenia: a systematic review and meta-analysis of 39 studies (N = 5151 participants). Nutr Rev.

[78] Vural H, Demirin H, Kara Y, Eren I, Delibas N (2010). Alterations of plasma magnesium, copper, zinc, iron and selenium concentrations and some related erythrocyte antioxidant enzyme activities in patients with Alzheimer’s disease. J Trace Elem Med Biol.

[79] Paoletti P, Vergnano AM, Barbour B, Casado M (2009). Zinc at glutamatergic synapses. Neuroscience.

[80] Sensi SL, Paoletti P, Bush AI, Sekler I (2009). Zinc in the physiology and pathology of the CNS. Nat Rev Neurosci.

[81] Toda M, Abi-Dargham A (2007). Dopamine hypothesis of schizophrenia: making sense of it all. Curr Psychiatry Rep.

[82] Li Y, Hasenhuetl PS, Schicker K, Sitte HH, Freissmuth M, Sandtner W (2015). Dual action of Zn^2+^ on the transport cycle of the dopamine transporter. J Biol Chem.

[83] Krall RF, Tzounopoulos T, Aizenman E (2021). The function and regulation of zinc in the brain. Neuroscience.

[84] Shen Z, Haragopal H, Li YV (2020). Zinc modulates synaptic transmission by differentially regulating synaptic glutamate homeostasis in hippocampus. Eur J Neurosci.

[85] Yamauchi T, Kamon J, Ito Y, Tsuchida A, Yokomizo T, Kita S (2003). Cloning of adiponectin receptors that mediate antidiabetic metabolic effects. Nature.

[86] Lorsch ZS, Hamilton PJ, Ramakrishnan A, Parise EM, Salery M, Wright WJ (2019). Stress resilience is promoted by a Zfp189-driven transcriptional network in prefrontal cortex. Nat Neurosci.

[87] Caracciolo V, Young J, Gonzales D, Ni Y, Flowers SJ, Summer R (2018). Myeloid-specific deletion of Zfp36 protects against insulin resistance and fatty liver in diet-induced obese mice. Am J Physiol Endocrinol Metab.

[88] Yuniati L, Scheijen B, van der Meer LT, van Leeuwen FN (2019). Tumor suppressors BTG1 and BTG2: beyond growth control. J Cell Physiol.

[89] Jiang H, Zhu Y, Zhou Z, Xu J, Jin S, Xu K (2018). PRMT5 promotes cell proliferation by inhibiting BTG2 expression via the ERK signaling pathway in hepatocellular carcinoma. Cancer Med.

[90] Micheli L, Ceccarelli M, Farioli-Vecchioli S, Tirone F (2015). Control of the normal and pathological development of neural stem and progenitor cells by the PC3/Tis21/Btg2 and Btg1 genes. J Cell Physiol.

[91] Miyata S, Mori Y, Tohyama M (2008). PRMT1 and Btg2 regulates neurite outgrowth of Neuro2a cells. Neurosci Lett.

