# Supplementary material for: The schizophrenia-associated missense variant rs13107325 regulates dendritic spine density
Source: Transl Psychiatry. 2022 Sep 2;12:361. doi: 10.1038/s41398-022-02137-z (PMC9440106; doi:10.1038/s41398-022-02137-z)
Supplement: Supplementary file 1 — Supplementary_Material [file 41398_2022_2137_MOESM1_ESM.docx]

**Supplementary for**

**The schizophrenia-associated missense variant rs13107325 regulates dendritic spine density**

Shiwu Li^1^, Changguo Ma^1^, Yifan Li^1^, Rui Chen^1,2^, Yixing Liu^1^, Li Pear Wan^2,3^, Qiuxia Xiong^4^, Chuang Wang^5^, Yongxia Huo^1^, Xinglun Dang^1,2^, Yongfeng Yang^6^, Luxian Lv^6^, Xi Chen^7^, Nengyin Sheng^3,*^, Wenqiang Li^6,*^ and Xiong-Jian Luo^8,9,*^

^1^Key Laboratory of Animal Models and Human Disease Mechanisms of the Chinese Academy of Sciences, Kunming Institute of Zoology, Chinese Academy of Sciences, Kunming, Yunnan, 650223, China.

^2^Kunming College of Life Science, University of Chinese Academy of Sciences, Kunming, Yunnan, 650204, China.

^3^State Key Laboratory of Genetic and Resources, Kunming Institute of Zoology, Chinese Academy of Sciences, Kunming, Yunnan, 650223, China.

^4^Department of Clinical Laboratory, the First Affiliated Hospital of Kunming Medical University, Kunming, Yunnan, 650032, China.

^5^Department of Pharmacology, and Provincial Key Laboratory of Pathophysiology in Ningbo University School of Medicine, Ningbo, Zhejiang, 315211, China.

^6^Henan Mental Hospital, the Second Affiliated Hospital of Xinxiang Medical University, Xinxiang, Henan 453002, China.

^7^First Department of Neurosurgery, The Second Affiliated Hospital of Kunming Medical University, Kunming, Yunnan, 650101, China.

^8^Zhongda Hospital, School of Life Sciences and Technology, Advanced Institute for Life and Health, Southeast University, Nanjing, Jiangsu, 210096, China.

^9^Department of Neurology, Affiliated Zhongda Hospital, Southeast University, Nanjing, Jiangsu, 210096, China.

Shiwu Li, Changguo Ma, Yifan Li, Rui Chen, Yixing Liu and Li Pear Wan contributed equally to this work.

Correspondence to: Xiong-Jian Luo, Zhongda Hospital, School of Life Sciences and Technology, Advanced Institute for Life and Health, Southeast University, Nanjing, Jiangsu 210096, China; Email: luoxiongjian@seu.edu.cn; or Wenqiang Li, Email: lwq781603@163.com; or Nengyin Sheng, Email: shengnengyin@mail.kiz.ac.cn.

**SUPPLEMENTARY METHODS**

***Generation of SLC39A8-p393T knock-in mice***

Protein sequence alignment (https://www.ebi.ac.uk/Tools/psa/) showed that human and mouse SLC39A8 share 93.3% sequence similarity, indicating that SLC39A8 is highly conservative in mouse and human (**Supplementary Figure 3**). The amino acid encodes by rs13107325 (human) is SLC39A8-p.Ala391Thr, which corresponds to mouse SLC39A8-p.393. The risk allele (T) of rs13107325 encodes threonine (T), while the protective allele (C) encodes alanine (Ala). Sequence analysis indicates that the amino acid corresponding to human rs13107325 in mouse SLC39A8-p.393 is Ala, we thus used CRISPR-Cas9 to generate a knock-in mouse model, which carried an amino acid replacement (i.e., Thr) at SLC39A8-p.393 (**Figure 1**). The SLC39A8-p.393T knock-in mice thus can model human SLC39A8 which contains the risk allele of rs13107325. Sanger sequencing validated that SLC39A8-p.393T was successfully introduced in the knock-in mouse model (**Figure 1**). The knock-in mice were generated by Shanghai Model organisms Center, Inc. (<https://www.modelorg.com/en/>) in October 2016. All mice were housed and maintained in SPF-level laboratory animal room (in Kunming institute of zoology, Chinese academy of sciences) at 22°C, with 60-65% humidity and 12 hours light/dark cycles (light: 8:00-20:00 and dark: 20:00-8:00), and were fed with regular sterilized rodent chow and water. Experimental mice were deeply anesthetized with isoflurane or CO_2_ before dissecting and isolating related tissues or biological samples. This study was approved by the Ethics Committee of Kunming Institute of Zoology, Chinese Academy of Sciences.

***Concentration measurements of cadmium, zinc, manganese and iron***

First, the brain tissues (or samples) were weighed and placed into 15 mL centrifuge tubes with 1 mL HNO_3_ and 0.5 ml H_2_O_2_ solution. The tubes were incubated in water bath at 60℃ to make the samples completely dissolved, then cooled to room temperature. Second, 0.10 mL 1.0 μg/mL cesium was added into the dissolved solutions as an internal standard, and 1% HNO_3_ was used (for a constant volume of 10 mL) and mixed thoroughly for inductively coupled plasma mass spectrometry (ICP-MS) analysis. Third, standard solutions containing zinc, manganese, iron and cadmium and working internal solutions of cesium were prepared from individual element standard stock solutions (obtained from Laboratory of the Government Chemist (LGC)). The concentration of zinc, manganese, iron and cadmium in the sample were measured by ICP-MS (Agilent, ICP-MS 7700, America). The parameters used in ICP-MS were summarized in **Supplementary Table 1**. The elements of zinc, manganese, iron and cadmium were detected at mass-to-charge ratio 65, 55, 56 and 112, respectively. A total of 8 mice were tested with double blinded (wild-type: n = 4; knock-in: n = 4).

***Proliferation and migration assays***

Brains were dissected on ice and were fixed with 4% PFA, then dehydrated with 30% sucrose and were embedded in Tissue-Tek O.C.T. compound (Cat. No: 4583, SAKURA) at −80 °C. The coronal sections with a thickness of 20 μm were prepared by freezing microtome (Thermo Scientific, CryoStar NX50) at −22 °C. The EdU positive cells were detected by cell-light EdU Apollo567 In Vitro Kit (Cat. No: C10310-1, RibBio) and the immunohistochemical staining were performed according to the manufacturer’s instructions. EdU positive cells were counted by using Image J software (https://imagej.nih.gov/ij/) and the mouse genotypes were validated by Sanger sequencing. The coronal sections were photographed by Laser scanning confocal microscope (OLYMPUS, FV1000) with 40 × under the oil lens. A total of 48 photographs from 8 fetal mice (E13.5) (knock-in: n = 4; wild-type: n = 4) were used for evaluating the proliferation of NSCs, and 30 photographs from 6 mice fetal mice (P0) (knock-in: n = 3; wild-type: n = 3) were used for evaluating the migration of NSCs.

***Transcriptome analysis and qPCR***

The cortical and hippocampal tissues were isolated from two or five-month-old mice. The tissues from two-month-old mice were used for RNA sequencing, and the tissues from five-month-old mice were used for qPCR. Then, the obtained tissues were freeze by liquid nitrogen and grinded into powders with a grinding rod. The total RNAs were extracted from tissue powders with TRIZOL reagent (Cat. No: 10296028, Invitrogen), and the cDNA libraries were prepared using the extracted RNA (from two-month-old mice) and the NEBNext® Ultra™ RNA Library Prep Kit for Illumina® (Cat. No: E7530L, NEB). RNA sequencing was conducted using the Illumina platform (HiSeq/X-ten) at Annoroad company (Beijing, China). The detailed processes of RNA-seq analysis were described in our previous study [1]. Briefly, hisat2 (version 2.1.0) [2] was used to map the clean reads to mouse genome (GRCm38) with default parameters, and Stringtie (version 1.3.4) [3] was used to assemble transcripts with default parameters. DEseq2 [4] in R (v3.5.1) was used to identify DEGs. Bonferroni method was conducted to correct *P* values. The DEGs was defined as *P*adj < 0.05. The *Actb*, *Slc39a8* and three DEGs, were detected by qPCR using PrimeScript™ RT reagent Kit with gDNA Eraser (Cat. No: RR047A, Takara), TB Green™ Premix Ex Taq™ II (Tli RNaseH Plus) (Cat. No: RR820A, Takara) and CFX96 Touch™ Real-Time PCR Detection System (Bio-Rad). The qPCR primers used in this study are presented in **Supplementary Table 2**.

***Spatio-temporal expression of Slc39a8***

The whole mouse brains were collected at E13.5, E15.5, E17.5, P0, P7, P14, P30 and P60 stages. qPCR was used to detect the *Actb* and *Slc39a8* mRNAs expression. The qPCR primers used in this study are presented in **Supplementary Table 2**.

***Behavioral and cognitive analysis***

Seven behavioral tests (open ﬁeld, light-dark transition, elevated plus maze, novel object recognition, Y maze, 3-chambered social approach and tail suspension) were performed between 9:00 a.m. and 19:00 p.m. in this study. The wild-type and knock-in mice were tested alternately to avoid the inﬂuence of circadian variances. Each behavioral test was performed at least an interval of 48 hours for minimizing the stress effects.

***Open ﬁeld test***

Open field test is mainly used to evaluate the locomotion and anxiety-like behavior of animals in novel environments [5]. The test was conducted in an open ﬁeld arena (length, width, height: 40 cm, 40 cm, 40 cm). The arena was virtually divided into sixteen same square and center and corner areas were set (**Supplementary Figure 5 A**). The tested mouse was placed in the center of the arena and allowed to freely explore the novel environment for 5 min. The movement trace of tested mouse in arena was recorded by video with SuperMaze software (Xinruan, China). The time and distance of tested mouse in the center and corner areas were evaluated by software with the same parameters. Before each trial, the open ﬁeld arena was cleaned with 30% ethanol. A total of 42 mice were tested successfully (wild-type: n = 20; knock-in: n = 22).

***Light-dark*** ***transition test***

The light-dark transition test is a useful method to study anxiolytic-like or anxiogenic-like activity of mouse based on the conflict between innate aversion to brightly illuminated areas and spontaneous exploratory behavior of rodents [6]. The test apparatus consists of two same size (length, width, height: 20 cm, 15 cm, 25 cm) compartments (a dark safe box and a brightly illuminated box) (**Supplementary Figure 5 G**). The test mouse was placed in the center of the brightly illuminated box and allowed to freely shuttle back and forth between the light and dark boxes for 10 min. The movement trace of mouse in the light box were recorded by video with SuperMaze software. The time and distance of test mouse in the light box were evaluated by software with the same parameters, and the transition times of test mouse were manually recorded. Before each trial, the light and dark boxes were cleaned with 30% ethanol. A total of 42 mice were tested successfully (wild-type: n = 20; knock-in: n = 22).

***Elevated plus maze test***

The elevated plus maze test is used to assess anxiety-related behavior in rodent model [7]. Rodents tend to avoid open and brightly illuminated places and explore new spaces, which results in anxiety-related behavior [7]. The elevated plus maze apparatus (which is approximately 55 cm above the ground) consists of 4 arms (two close arms (length, width, height: 30 cm, 6 cm, 15 cm) and two open arms (length, width: 33 cm, 8 cm) in cross shape with central zone (length, width: 6 cm, 8 cm) in the middle (**Supplementary Figure 5 L**). The elevated plus maze test was conducted as previously described [7]. Briefly, the test mouse was placed on the open arm and allowed to freely explore the plus maze for 5 mins. The movement traces were recorded by video with SuperMaze software, and the times spent in open arm were evaluated by software with the same parameters. The elevated plus maze was cleaned with 30% ethanol after the tested mouse accomplished the test. A total of 42 mice were tested successfully (wild-type: n = 20; knock-in: n = 22).

***Novel object recognition test***

Novel object recognition test is used to evaluate the abilities of learning and memory in mice, particularly recognition memory [8]. The spontaneous tendency of rodents made them spend more time on exploring a novel object than a familiar one. Exploring novel object reflects the use of learning and recognition memory in rodents. Novel object recognition test were conducted as previously described [8]. Briefly, the test processes contain three phrases, including habituation phrase, training phrase and testing phrase with 24 hours intertrial intervals (ITIs) (**Supplementary Figure 6 A**). During habituation phrase, the tested mouse was allowed to freely explore the open field arena (length, width, height: 40 cm, 40 cm, 40 cm) for 5 min. During training phrase, the tested mouse was allowed to freely explore the arena with 2 identical objects placed along the diagonal for 10 min. During testing phrase, one of two identical objects was replaced by one novel object, then the tested mouse was allowed to freely explore the arena with two different objects (a familiar object and a novel object) for 10 min. The open field and objects were cleaned with 30% ethanol after the test mouse accomplished each phrase. Behaviors of each mouse were recorded by video with Supermaze software and the time of exploration on two different objects were evaluated by software with the same parameters. A total of 40 mice were tested successfully (wild-type: n = 19; knock-in: n = 21).

***Y maze test***

Y-maze is mainly used to study spatial working memory in mice based on the nature of mice to explore the unknown environment [9]. The test was conducted in an “Y” style geometric arena (one arm length, width, height: 35 cm, 5 cm, 15 cm) which has three arms defined as A, B, and C areas (**Supplementary Figure 6 D**). Y maze test was conducted as previously described [10]. Briefly, the test mouse was placed in the arm of A and allowed to freely explore apparatus for 8 mins. The mice activity in apparatus was recorded by video with Supermaze software and the series of arm entries were recorded manually. The event that if the consecutive three arm entries are different can be defined as a spontaneous alternation. For example, the movement trace of the mouse from A arm to B arm and then to C arm can be counted for one spontaneous alternation. Before each trial, the Y maze apparatus was cleaned with 30% ethanol. A total of 42 mice were tested successfully (wild-type: n = 20; knock-in: n = 22).

***3-chambered social test***

The three-chamber social test is used to assess the sociability and preference for social novelty in mouse [11]. The social testing apparatus consists of three-chambered box (each chambered box length, width and height are: 40 cm, 20 cm, 22 cm) (**Supplementary Figure 7A**). The three-chamber social test was conducted as previously described [12]. Briefly, the test process contains three sessions with no ITIs, each session costs 10 mins. In the first session, the test mouse was allowed to habituate to the apparatus and to freely explore the three chambers. In the second session, a stranger C57BL/6J male mouse (stranger 1) was placed in stainless steel pencil cup at left side chamber and the same cup at right side chamber was empty. In the third session, a novel stranger C57BL/6J male mouse (stranger 2) was placed in cub at right side of the chamber. The test mouse was replaced in the middle chamber after placing the stranger 1 or 2 into the test apparatus. The chambers and cups were cleaned with 30% ethanol after the test mouse accomplishing these three sessions. Behaviors were recorded by video with Supermaze software and the time spent sniffing each cup were evaluated by software under the same parameters. A total of 40 mice were tested successfully (wild-type: n = 20; knock-in: n = 20).

***Tail suspension test***

The suspension tail test is used to assess the depressive behavior of mice, based on a normal animal submitted to an insoluble, aversive situation alternates the agitation and immobility behaviors [13]. The test was conducted as previously described [14,15]. Briefly, the mouse tail was fixed on the vertical stick which fixed in the box (length, width, height: 20 cm, 20 cm, 32 cm) and the mouse body was hung down in the air at the specific height for 6 mins (**Supplementary Figure 7D**). Behaviors were recorded by video with SuperTst software and the behaviors data were evaluated by software with the same parameters in the last 4 mins. The immobile state of test mice was evaluated by software under the same parameters, and the immobile state (such as, limbs motionless, no climbing, swaying passively) was defined by the activity of test mice. A total of 37 mice were tested successfully and three mice were drop down during the test (wild-type: n = 19; knock-in: n = 18).

***Analysis of dendritic spine density***

Briefly, the brains were dissected and immersed into a mixture solution containing equal volumes of solution A and B, and incubated at room temperature for two weeks in dark environment. The brains were then transferred into solution C. After incubating for one week (in dark), the brains were embedded with Tissue-Tek O.C.T. compound (Cat. No: 4583, SAKURA) at -80 ℃. Coronal sections (100 μm) were prepared by a freezing microtome (Thermo Scientific, CryoStar NX50, America), and transferred to gelatin (Cat. No: 3606C435, Amresco) coated slides (Dropping a small amount of FD solution C on the sections with poor adhesion). After drying the sections at room temperature (in dark) for 12 hours, sections were stained using solutions D and E. Then, the stained sections were dehydrated with 50% (one time, 4 min each time), 75% (one time, 4 min each time) and 95% (five times, 4 min each time) ethanol sequentially, and hyalinized using xylene (three times, 4 min each time) in fuming cupboard. Finally, the prepared sections were covered with coverslips and Fluoromount-G (Cat. No: 0100-01, SouthernBiotech).

Six independent coronal sections containing cortex (including motor cortex and somatosensory cortex) and hippocampus from each mouse were selected to image the dendrite spines in cortex and hippocampus region, and three wild-type and three knock-in mice were used. The dendrite spines from cortex or hippocampus were randomly photographed by Laser scanning confocal microscope (OLYMPUS, FV1000) with 100× under the oil lens. The target dendrites were selected to take 1-3 pictures on Z-axis through manually adjusting the focal length. The serial pictures were merged using Photoshop software and at least 20 μm target dendrite was selected for dendrite spine density analysis. The Image J software (https://imagej.nih.gov/ij/) was used to measure the length of the dendrites and count the number of dendritic spines (double blinded). The criteria of dendritic spine and statistical method were referenced to previously study [16-18]. The number of dendrites in each mouse included in this study is provided in **Supplementary Table 3.** One-tailed *Student’s* t-test was used for statistical analysis. *P* < 0.05 was set as the threshold of significance.

**Supplementary Tables**

**Supplementary Table 1. Main operating parameters for ICP-MS 7700**

| **Parameters** | **Value** |
| --- | --- |
| **Plasma parameters** | |
| Rf power | 1.5 KW |
| Auxiliary gas ﬂow rate | 1.0 L/min |
| Carrier gas ﬂow rate | 1.0 L/min |
| Plasma gas flow | 15 L/min |
| Compensating gas flow | 0.1 L/min |
| **Ion lens parameters** | |
| Extract 1 | -140 V |
| Extract 2 | 4 V |
| Omega Bias | -30 V |
| Plate Bias | -31 V |
| QP Focus | 2 V |
| **Measure parameters** | |
| Resolution | 0.7±0.1 u |
| Measuring mode | Peak jumping |
| Measurement point/peak | 3 |
| Integration time | 100 ms |
| Sample lift | 0.2 mL/min |

**Supplementary Table 2. The primers used in this study**

| Primers | Sequences (5’>3’) |
| --- | --- |
| Mouse *Btg2*-qPCR-F: | ATGAGCCACGGGAAGAGAAC |
| Mouse *Btg2*-qPCR-R: | GCCCTACTGAAAACCTTGAGTC |
| Mouse *Zfp189*-qPCR-F: | TGATGGACAGCTACGGAAAGC |
| Mouse *Zfp189*-qPCR- R: | CGCTACCCACAGGATCTTGAT |
| Mouse *Has2*-qPCR-F: | TGTGAGAGGTTTCTATGTGTCCT |
| Mouse *Has2*-qPCR- R: | ACCGTACAGTCCAAATGAGAAGT |
| Mouse *Slc39a8*-qPCR-F: | GCCAAGCTCATGTACCTGTCT |
| Mouse *Slc39a8*-qPCR-R: | AAGATGCCCCAATCGCCAA |
| Mouse *Actb*-qPCR-F: | GGCTGTATTCCCCTCCATCG |
| Mouse *Actb*-qPCR-R: | CCAGTTGGTAACAATGCCATGT |

**Supplementary Table 3. The number of dendrites (from each mouse) included in this study**

| Cortex | | Hippocampus | |
| --- | --- | --- | --- |
| Groups | Number dendrites | Groups | Number dendrites |
| WT-1 | 12 | WT-1 | 24 |
| WT-2 | 28 | WT-2 | 35 |
| WT-3 | 36 | WT-3 | 30 |
| MT-1 | 22 | MT-1 | 24 |
| MT-2 | 35 | MT-2 | 35 |
| MT-3 | 37 | MT-3 | 31 |

**Supplementary Figures**


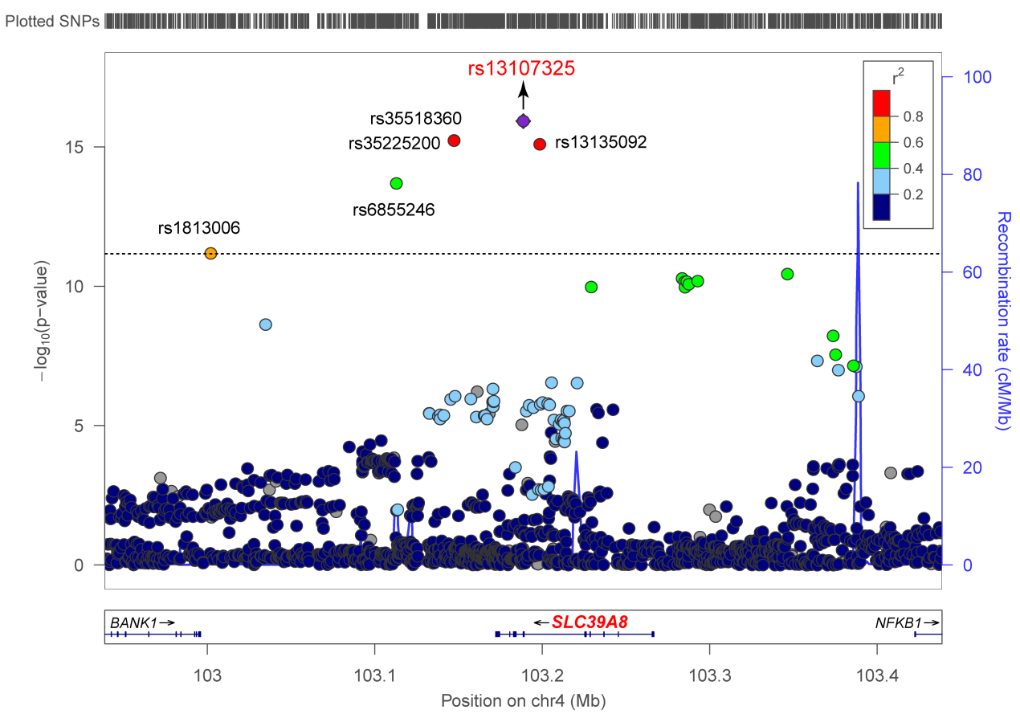


**Supplementary Figure 1. rs13107325 showed the most significant association with schizophrenia.** (Data were obtained from PGC+CLOZUK) [19].


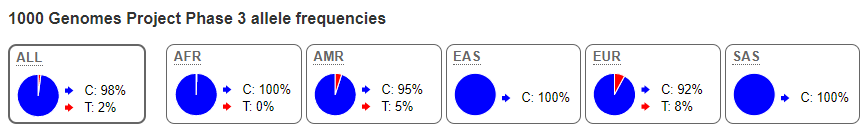


**Supplementary Figure 2. The frequency of the risk allele of rs13107325 in world populations**

All, all phase 3 individuals; AFR, African; AMR, American; EAS, East Asian; SAS, South Asian.

Data downloaded from Ensembl website: http://www.ensembl.org/index.html.


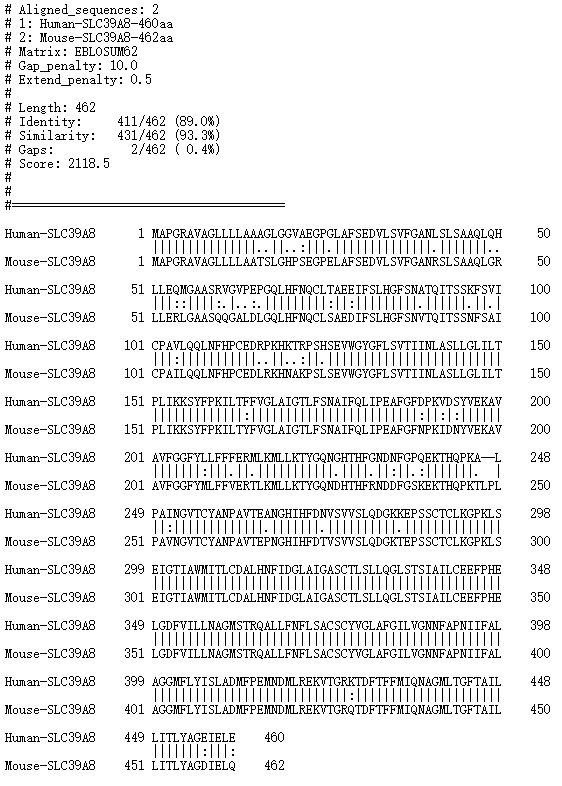


**Supplementary Figure 3. SLC39A8 protein is highly conserved in mouse and human.**

(Pairwise Sequence Alignment: https://www.ebi.ac.uk/Tools/psa/).


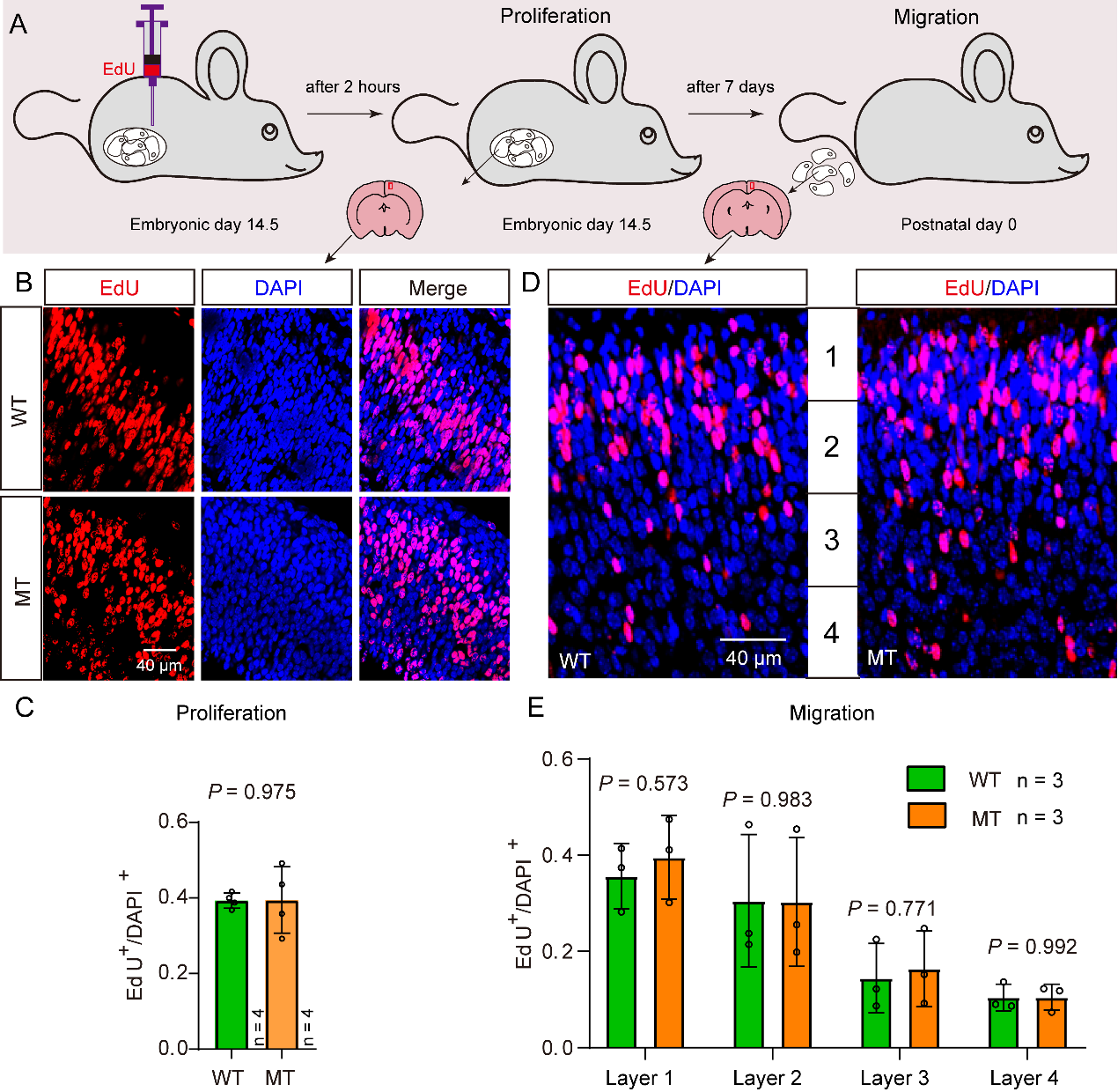


**Supplementary Figure 4. SLC39A8-p.393T did not affect proliferation and migration of neural stem cells. (A)** Overview of *in vivo* proliferation and migration assays. **(B-E)** The proliferation and migration of NSCs did not show significant differences in SLC39A8-p.393T knock-in mice and wild-types. (**B** and **D**) Representative immunofluorescence images for EdU staining. Two-tailed *Student’s* *t***-**test was used for statistical test. Data represent *mean ± SD*, *n* = 4 for **(C),** *n* = 3 for **(E)**.


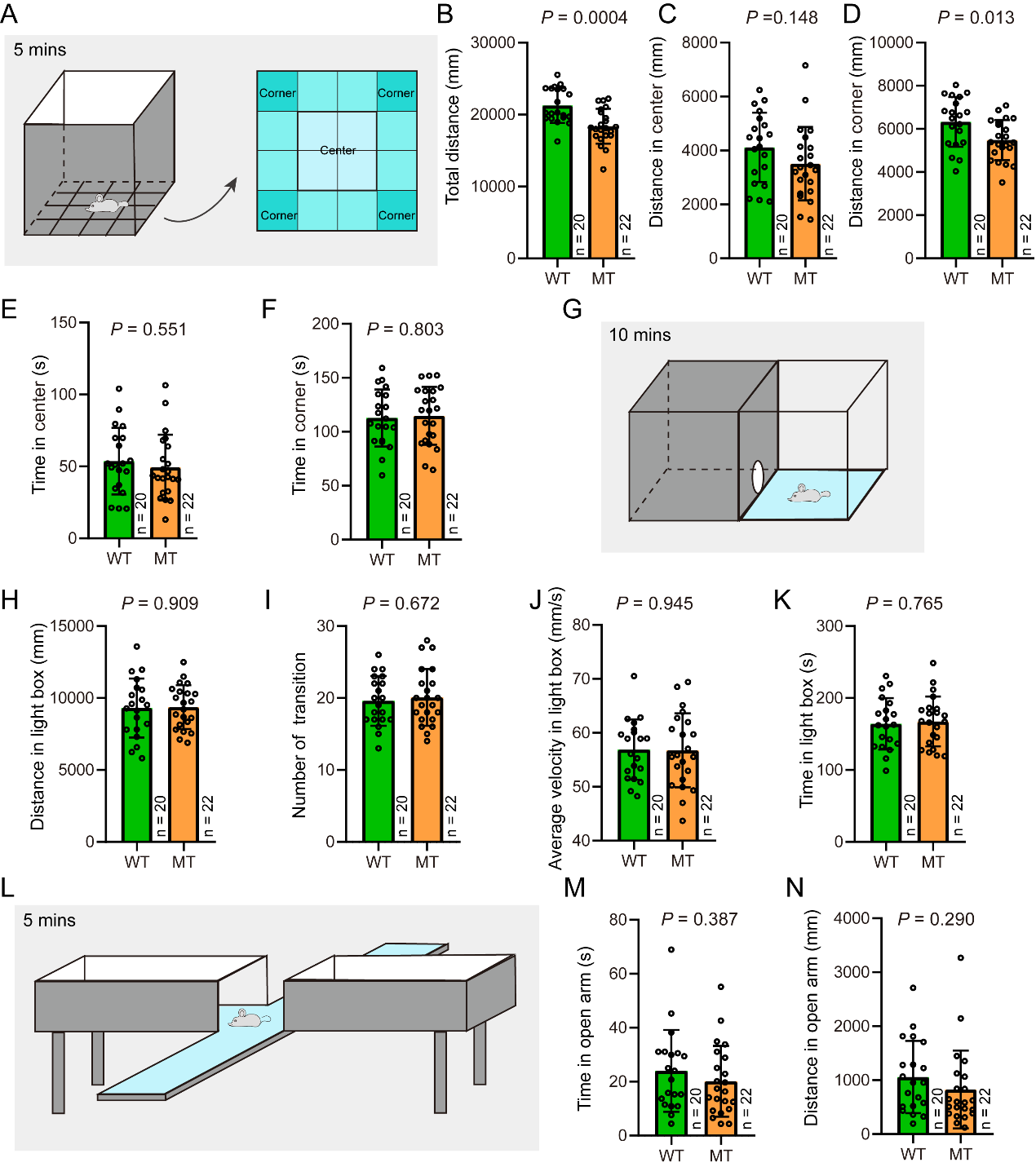
**Supplementary Figure 5. SLC39A8-p.393T mice showed no differences in anxiety-related behaviors compared with** **wild-types. (A)** Overview of open ﬁeld test. **(B-F)** The total distance and distance traveled in corner of SLC39A8-p.393T knock-in mice were significant decreased compared with wild-types. **(G)** Overview of light-dark transition test. (**H**-**K**) The distance in light box, number of transitions, average velocity in light box and time in light box did not show significant differences in SLC39A8-p.393T knock-in mice and wild-types. **(L)** Overview of elevated plus maze test. (**M**-**N**) The distance and time spent in open arm were not significantly different between SLC39A8-p.393T knock-in mice and SLC39A8-p.393A wild-type mice. Two-tailed *Student’s* *t***-**test was used for statistical test. Data represent *mean ± SD*, *n* (WT) = 20 and *n* (MT) = 22 for **(B-F)** and (**H**-**K**) and (**M**-**N**).


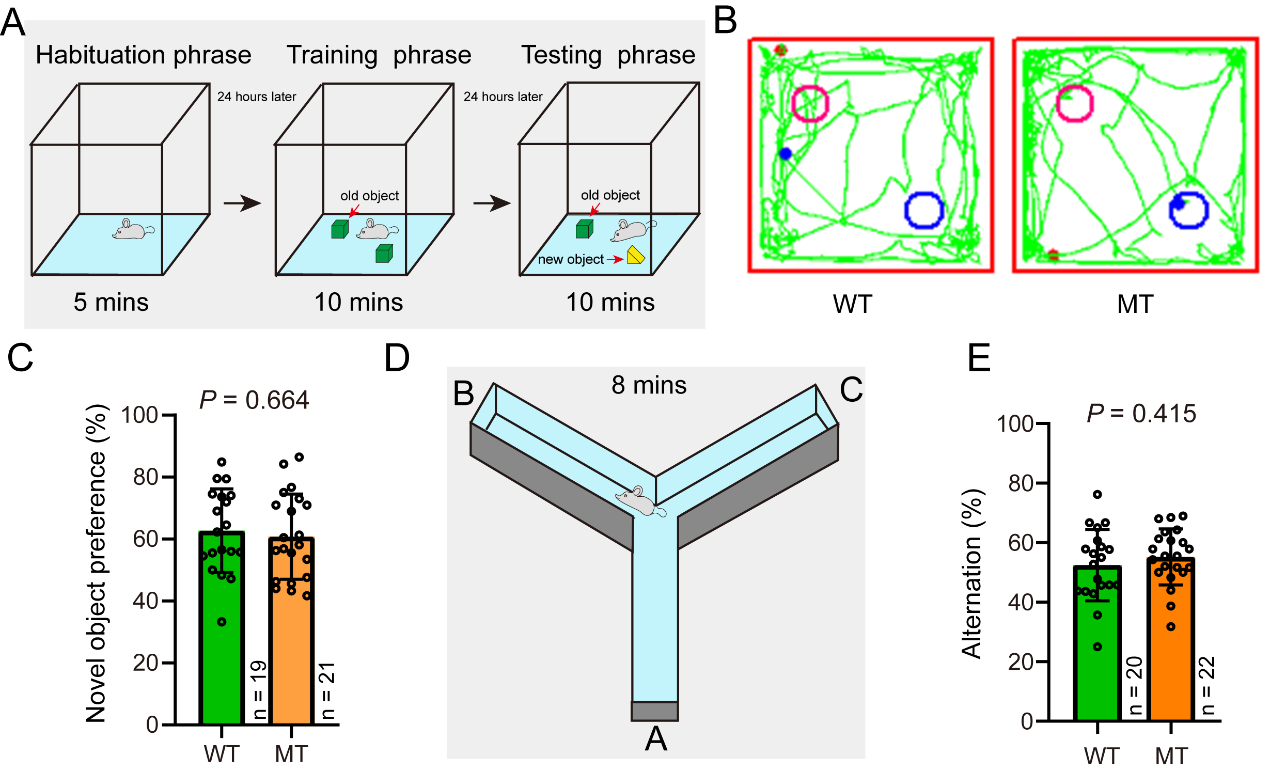


**Supplementary Figure 6. SLC39A8-p.393T mice and wild-types showed no differences in learning and memory-related behaviors. (A)** Overview of novel object recognition test. **(B)** Representative movement traces of the knock-in and wild-type mice for novel object recognition test. **(C)** There was no difference in novel object preference in SLC39A8-p.393T knock-in mice and SLC39A8-p.393A wild-type mice. **(D)** Overview of Y maze test. **(E)** SLC39A8-p.393T knock-in mice did not show difference in spontaneous alternation compared with SLC39A8-p.393A wild-type mice. Two-tailed *Student’s* *t***-**test was used for statistical test. Data represent *mean ± SD*, *n* (WT) = 19 and *n* (MT) = 21 for **(C)**, *n* (WT) = 20 and *n* (MT) = 22 for **(E)**.


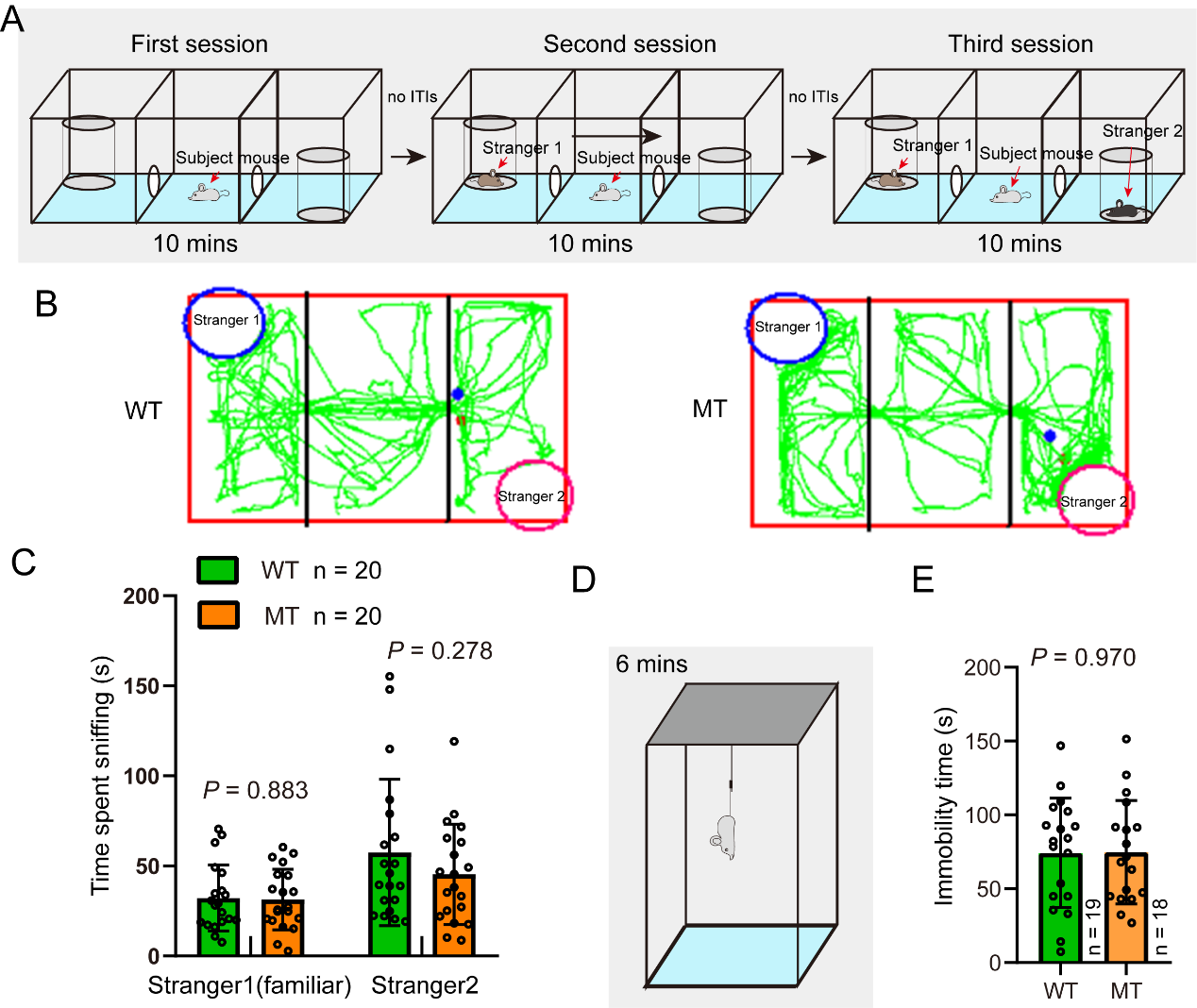


**Supplementary Figure 7. SLC39A8-p.393T and wild-type mice did not show difference in social preference and depressive behaviors. (A)** Overview of 3-chambered social test. **(B)** Representative movement traces of knock-in and wild-type mice for 3-chambered social test. **(C)** No difference in time spent sniffing in between SLC39A8-p.393T knock-in mice and SLC39A8-p.393A wild-type mice. **(D)** Overview of tail suspension test. **(E)** The immobility time did not show difference in SLC39A8-p.393T knock-in mice and SLC39A8-p.393A wild-type mice. Two-tailed *Student’s* *t***-**test was used for statistical test. Data represent *mean ± SD*, *n* (WT) = 20 and *n* (MT) = 20 for **(C)**, *n* (WT) = 19 and *n* (MT) = 18 for **(E)**.


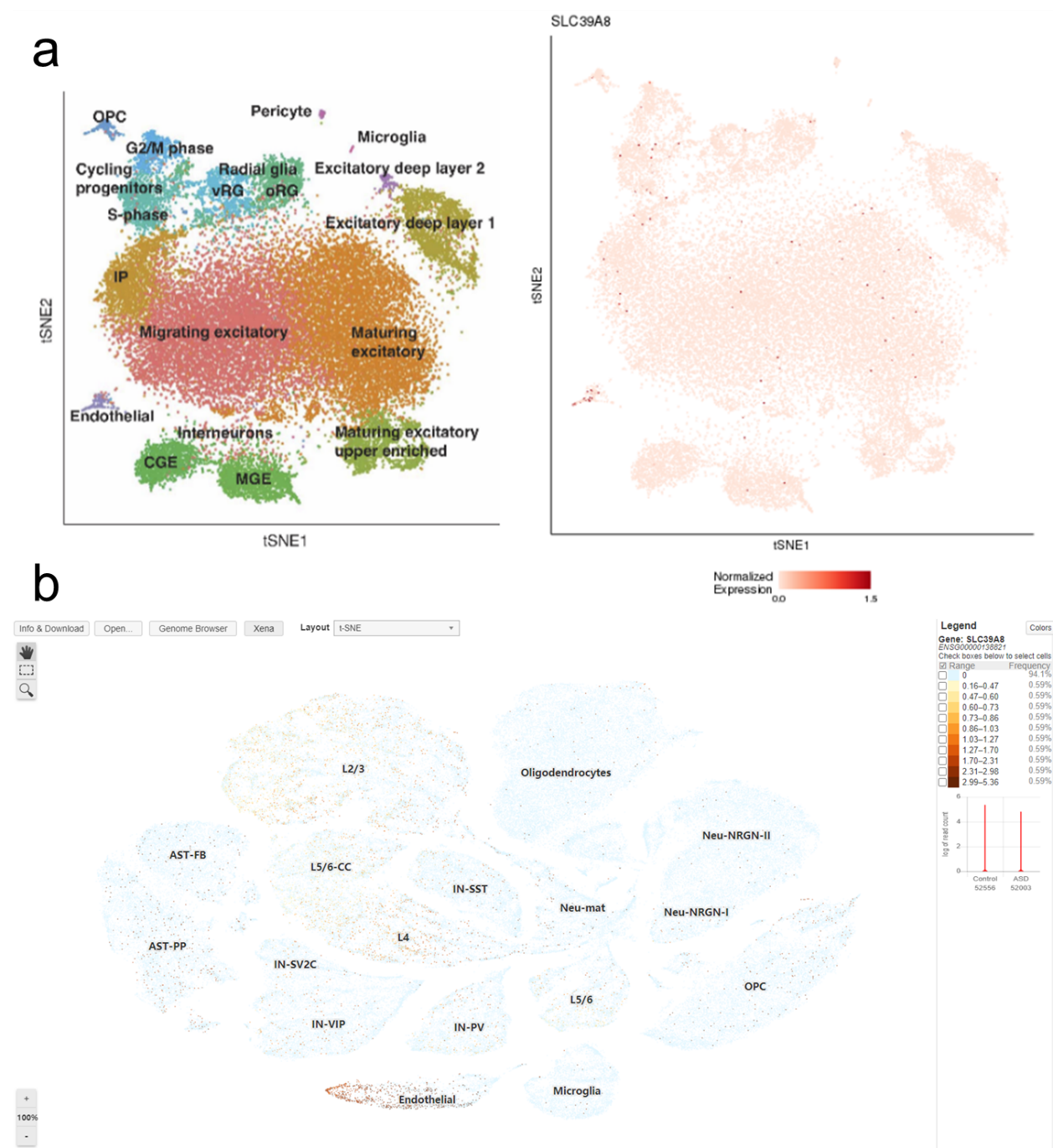


**Supplementary Figure 8. *SLC39A8* mRNA is mainly expressed in endothelial cells of human brain.** Data from CoDEx Viewer: <http://solo.bmap.ucla.edu/shiny/webapp/> and UCSC cell browser: <https://cells.ucsc.edu/?ds=autism>.

**
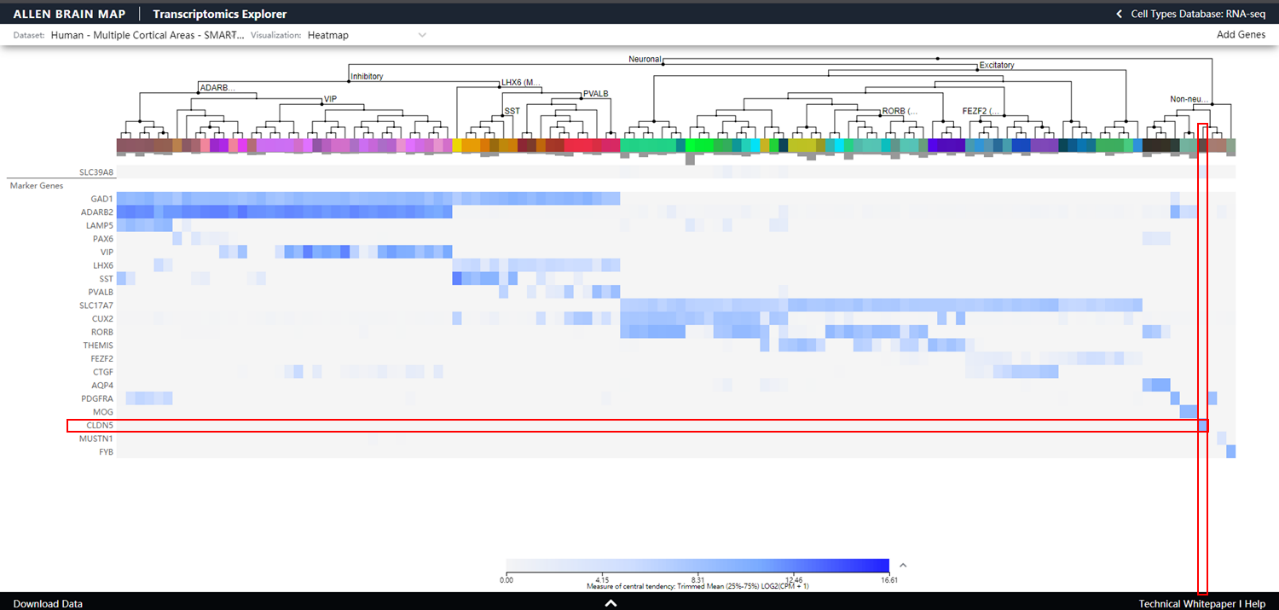
**

**Supplementary Figure 9. *SLC39A8* mRNA is mainly expressed in** **layer 2-5 CLDN5^+^ endothelial cells of human brain.** Data from of Transcriptomics Explorer of Allen brain map: <https://celltypes.brain-map.org/rnaseq/human_ctx_smart-seq?selectedVisualization=Heatmap&colorByFeature=Cell+Type&colorByFeatureValue=GAD>1


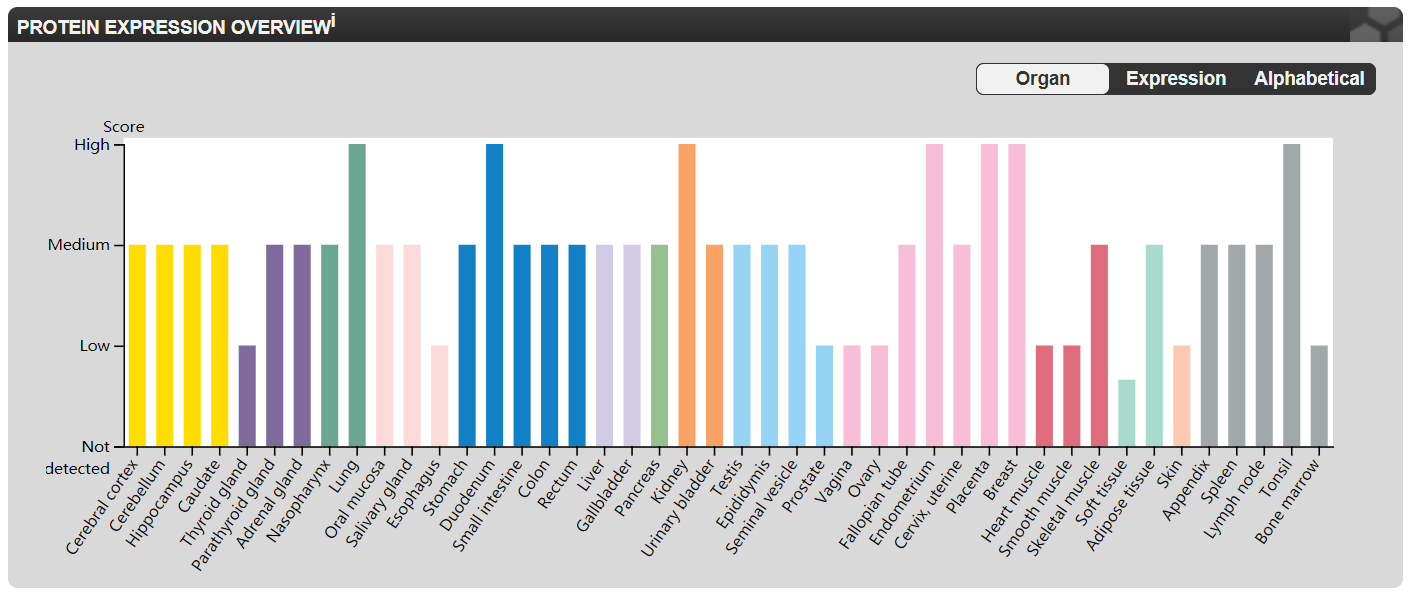


**Supplementary Figure 10. SLC39A8 protein is widely expressed in diverse human tissues.** (Data were from the Human Protein Atlas, https://www.proteinatlas.org/).


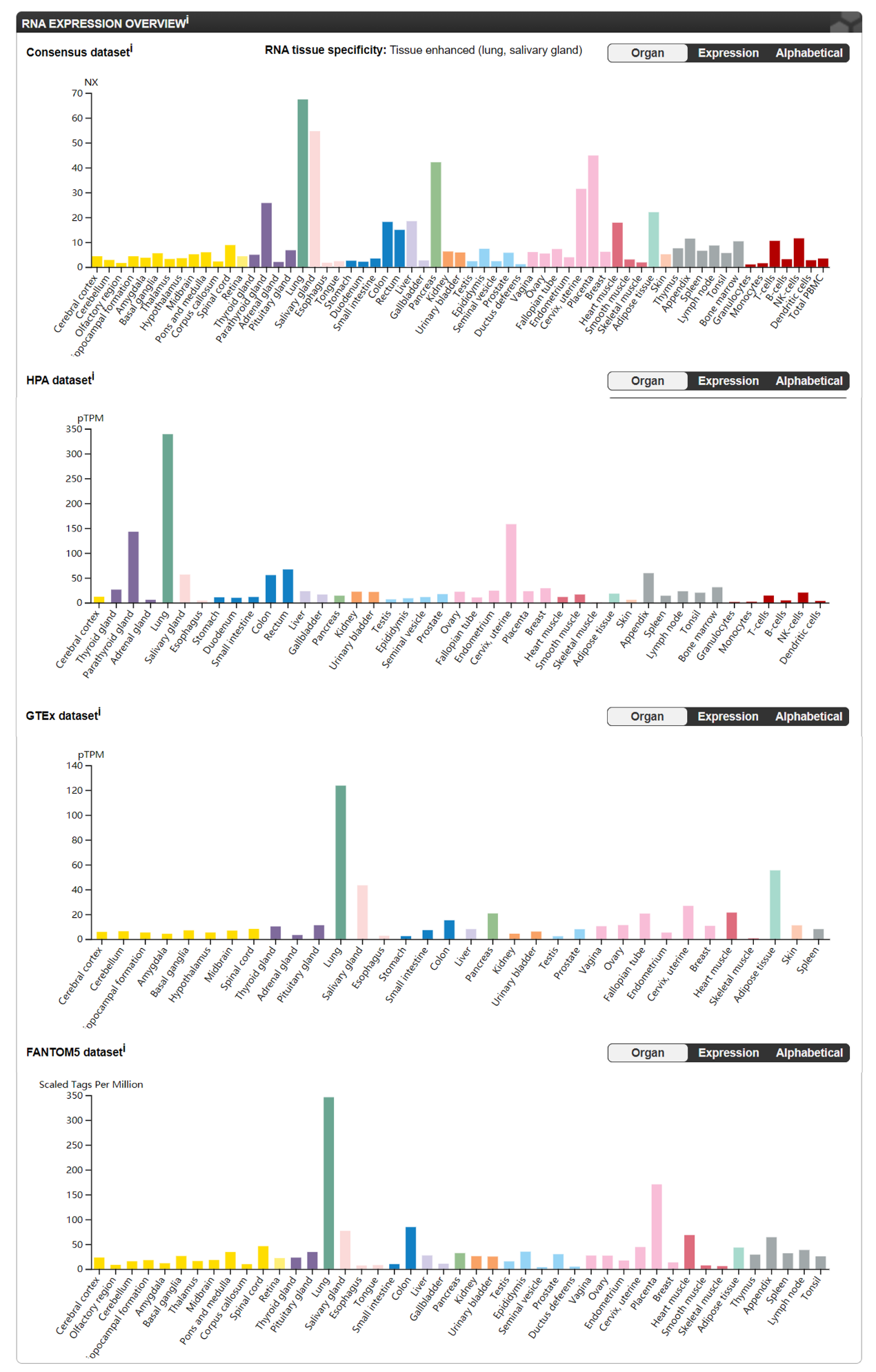


**Supplementary Figure 11. *SLC39A8* mRNA is widely expressed in diverse human tissues.**

(Data were from the Human Protein Atlas, https://www.proteinatlas.org/).


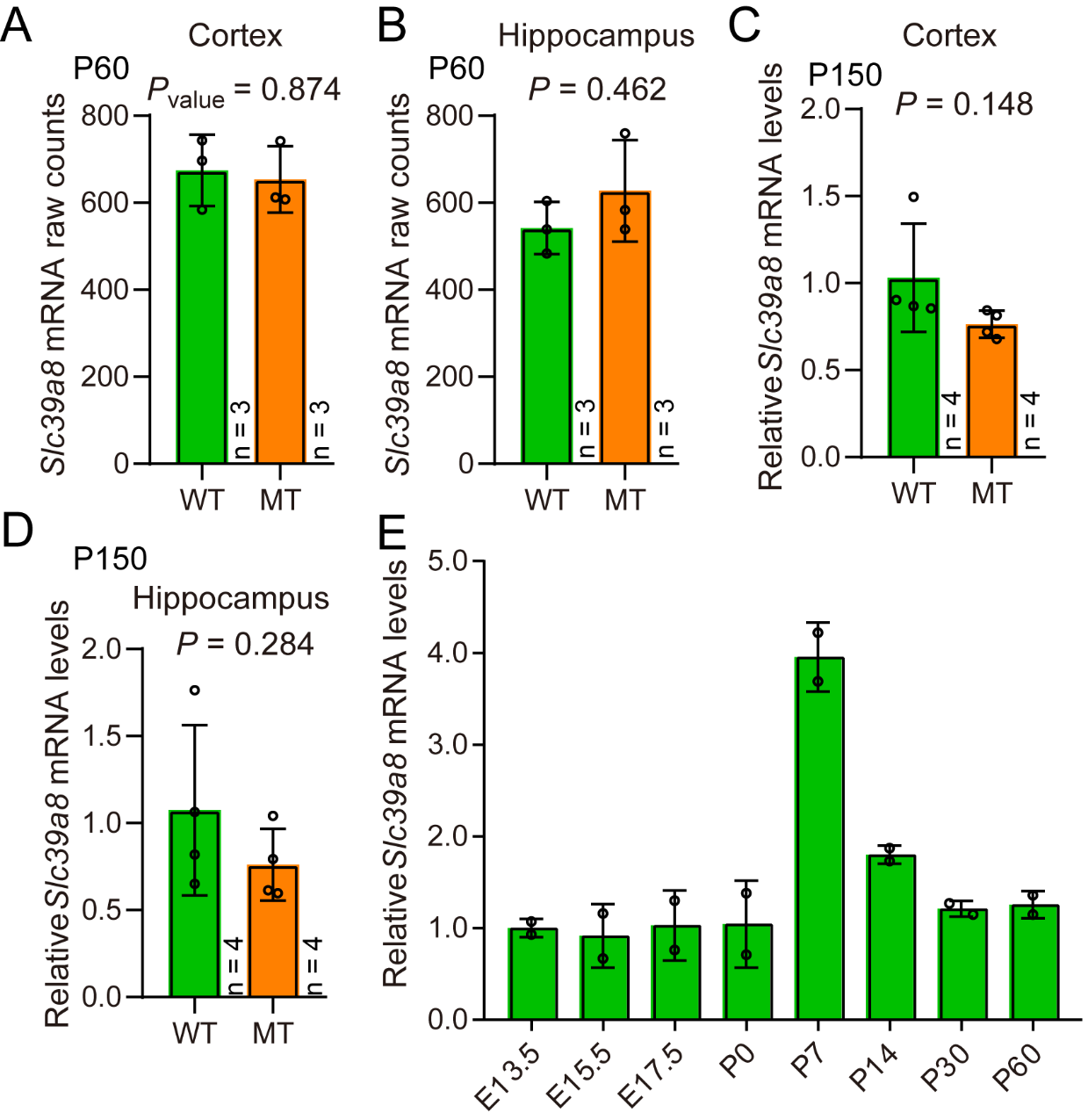


**Supplementary Figure 12. SLC39A8-p.393T knock-in mice did not affect *Slc39a8* mRNA expression. (A, B)** The RNA-seq data revealed that *Slc39a8* mRNA expression in cortex (A) and hippocampus (B) did not show difference between SLC39A8-p.393T knock-in mice and wild-type mice. **(C, D)** The *Slc39a8* mRNA expression in cortex (C) and hippocampus (D) did not show difference in SLC39A8-p.393T knock-in mice and wild-type mice. **(E)** The spatio-temporal expression of *Slc39a8* gene in mouse brain. Expression of *Slc39a8* in 8 developmental stages, E13.5, E15.5, E17.5, P0, P7, P14, P30 and P60, were quantified.

**REFERENCES**

1. Li K, Li Y, Wang J, Huo Y, Huang D, Li S, et al. A functional missense variant in ITIH3 affects protein expression and neurodevelopment and confers schizophrenia risk in the Han Chinese population*.* J Genet Genomics. 2020;47:233-48.

2. Kim D, Langmead B, Salzberg SL. HISAT: a fast spliced aligner with low memory requirements*.* Nat Methods. 2015;12:357-60.

3. Pertea M, Pertea GM, Antonescu CM, Chang TC, Mendell JT, Salzberg SL. StringTie enables improved reconstruction of a transcriptome from RNA-seq reads*.* Nat Biotechnol. 2015;33:290-5.

4. Love MI, Huber W, Anders S. Moderated estimation of fold change and dispersion for RNA-seq data with DESeq2*.* Genome Biol. 2014;15:550.

5. Kraeuter AK, Guest PC, Sarnyai Z. The Open Field Test for Measuring Locomotor Activity and Anxiety-Like Behavior*.* Methods Mol Biol. 2019;1916:99-103.

6. Bourin M, Hascoet M. The mouse light/dark box test*.* Eur J Pharmacol. 2003;463:55-65.

7. Belovicova K, Bogi E, Csatlosova K, Dubovicky M. Animal tests for anxiety-like and depression-like behavior in rats*.* Interdiscip Toxicol. 2017;10:40-3.

8. Lueptow LM. Novel Object Recognition Test for the Investigation of Learning and Memory in Mice*.* J Vis Exp. 2017.

9. Kraeuter AK, Guest PC, Sarnyai Z. The Y-Maze for Assessment of Spatial Working and Reference Memory in Mice*.* Methods Mol Biol. 2019;1916:105-11.

10. Rice J, Coutellier L, Weiner JL, Gu C. Region-specific interneuron demyelination and heightened anxiety-like behavior induced by adolescent binge alcohol treatment*.* Acta Neuropathol Commun. 2019;7:173.

11. Moy SS, Nadler JJ, Perez A, Barbaro RP, Johns JM, Magnuson TR, et al. Sociability and preference for social novelty in five inbred strains: an approach to assess autistic-like behavior in mice*.* Genes Brain Behav. 2004;3:287-302.

12. Faizi M, Bader PL, Saw N, Nguyen T-VV, Beraki S, Wyss-Coray T, et al. Thy1-hAPP(Lond/Swe+) mouse model of Alzheimer's disease displays broad behavioral deficits in sensorimotor, cognitive and social function*.* Brain and behavior. 2012;2:142-54.

13. Steru L, Chermat R, Thierry B, Simon P. The tail suspension test: A new method for screening antidepressants in mice*.* Psychopharmacology. 1985;85:367-70.

14. Can A, Dao DT, Terrillion CE, Piantadosi SC, Bhat S, Gould TD. The tail suspension test*.* J Vis Exp. 2012e3769.

15. Can A, Blackwell RA, Piantadosi SC, Dao DT, O'Donnell KC, Gould TD. Antidepressant-like responses to lithium in genetically diverse mouse strains*.* Genes, Brain and Behavior. 2011;10:434-43.

16. Risher WC, Ustunkaya T, Singh Alvarado J, Eroglu C. Rapid Golgi analysis method for efficient and unbiased classification of dendritic spines*.* PLoS One. 2014;9:e107591.

17. Dumanis SB, Cha HJ, Song JM, Trotter JH, Spitzer M, Lee JY, et al. ApoE receptor 2 regulates synapse and dendritic spine formation*.* PLoS One. 2011;6:e17203.

18. Tian Y, Tang FL, Sun X, Wen L, Mei L, Tang BS, et al. VPS35-deficiency results in an impaired AMPA receptor trafficking and decreased dendritic spine maturation*.* Mol Brain. 2015;8:70.

19. Pardiñas AF, Holmans P, Pocklington AJ, Escott-Price V, Ripke S, Carrera N, et al. Common schizophrenia alleles are enriched in mutation-intolerant genes and in regions under strong background selection*.* Nature genetics. 2018;50:381-9.
